# Combination Therapies for Biofilm Inhibition and Eradication: A Comparative Review of Laboratory and Preclinical Studies

**DOI:** 10.3389/fcimb.2022.850030

**Published:** 2022-02-25

**Authors:** Sophia Hawas, Anthony D. Verderosa, Makrina Totsika

**Affiliations:** ^1^ Centre for Immunology and Infection Control, Queensland University of Technology, Brisbane, QLD, Australia; ^2^ School of Biomedical Sciences, Queensland University of Technology, Brisbane, QLD, Australia

**Keywords:** antimicrobial resistance (AMR), anti-biofilm, infection, nitroxides, antibiotics, nitric oxide (NO), quorum sensing inhibitors (QSI), antimicrobial peptides (AMPs)

## Abstract

Microbial biofilms are becoming increasingly difficult to treat in the medical setting due to their intrinsic resistance to antibiotics. To combat this, several biofilm dispersal agents are currently being developed as treatments for biofilm infections. Combining biofilm dispersal agents with antibiotics is emerging as a promising strategy to simultaneously disperse and eradicate biofilms or, in some cases, even inhibit biofilm formation. Here we review studies that have investigated the anti-biofilm activity of some well-studied biofilm dispersal agents (e.g., quorum sensing inhibitors, nitric oxide/nitroxides, antimicrobial peptides/amino acids) in combination with antibiotics from various classes. This review aims to directly compare the efficacy of different combination strategies against microbial biofilms and highlight synergistic treatments that warrant further investigation. By comparing across studies that use different measures of efficacy, we can conclude that treating biofilms *in vitro* and, in some limited cases *in vivo*, with a combination of an anti-biofilm agent and an antibiotic, appears overall more effective than treating with either compound alone. The review identifies the most promising combination therapies currently under development as biofilm inhibition and eradication therapies.

## Introduction

When planktonic bacterial cells contact a surface, whether biotic or abiotic, they can irreversibly attach to it, proliferate, and form complex three-dimensional communities known as biofilms (Vestby et al., 2020). Biofilms are inherently tolerant to environmental stress, antimicrobials, and host immune responses (McDougald et al., 2011; Vestby et al., 2020). Consequently, biofilms pose a major clinical challenge as biofilm-related infections are extremely difficult to treat or permanently eradicate, with very few viable treatments or management options available (Azevedo et al., 2020; Vishwakarma, 2020). Current strategies for managing biofilm-related infections are aimed at: (i) preventing biofilm formation, (ii) limiting biofilm expansion, or (iii) biofilm eradication by chemical or mechanical means (e.g. removal) (Vuotto and Donelli, 2019; Zhao et al., 2019 Azevedo et al., 2020).

Achieving complete biofilm eradication with antibiotics alone is extremely challenging, with even clinically significant bacterial reduction being often hard to achieve. The precise mechanism by which conventional antibiotics fail to eradicate biofilms is not fully understood and has been the topic of extensive investigation (Gilbert et al., 2002; Lewis, 2008; Høiby et al., 2010). Restricted drug penetration resulting from the presence of a protective extracellular matrix, reduced cell growth, and the presence of persister cells within biofilms (quiescent cells exhibiting extreme antimicrobial tolerance) are all thought to contribute to the high antibiotic tolerance of biofilms (McDougald et al., 2011). To address this challenge, several new and innovative strategies have come under intense investigation (Barraud et al., 2015; Brackman and Coenye, 2015; Pletzer and Hancock, 2016; Fleming and Rumbaugh, 2017). Many of these, such as the development of biofilm inhibition, dispersal, and eradication agents, have been extensively reviewed elsewhere (Fleming and Rumbaugh, 2017; Roy et al., 2018; Verderosa et al., 2019c; Ghosh et al., 2020). Here we focus on studies whereby biofilm dispersal agents are co-administered with antimicrobials and evaluated as combination treatment strategies against bacterial biofilms.

**Figure 1:**
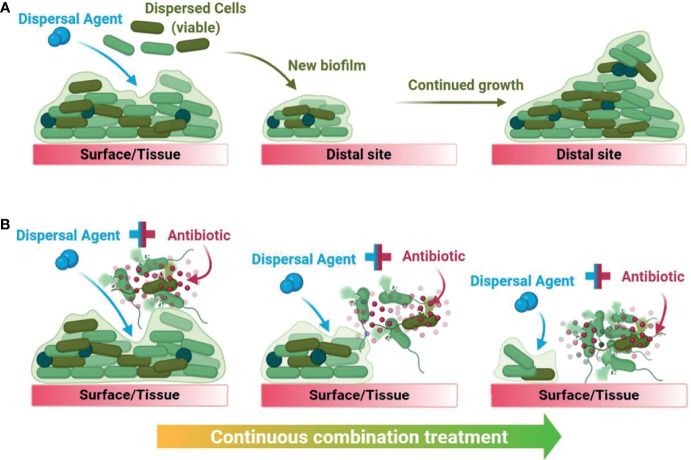
Graphical summary of the downstream consequences of treating a biofilm with dispersal agents alone **(A)** versus with a biofilm dispersal-eradication combination strategy **(B)**, demonstrating how it is clinically and industrially relevant.

Biofilm dispersal agents rarely possess inherent antimicrobial activity. As such, their potential use as biofilm treatment strategies requires supplementation with an effective antimicrobial agent to successfully disperse and eradicate biofilms (Fleming and Rumbaugh, 2018). Several promising classes of biofilm dispersal agents have been reported to date (Kaplan, 2010); however, the true therapeutic potential of dispersal agents lies in their ability to restore or synergistically enhance the activity of commonly prescribed antimicrobials. In clinical settings, co-treatment is imperative as biofilm dispersal alone would result in translocation of live bacteria to new sites in the body and the subsequent seeding of new infection foci (Fleming and Rumbaugh, 2018). Upon combining a dispersal agent with an effective antibiotic/antimicrobial, the combined treatment both disperses and eradicates biofilm-residing cells, thus preventing further dissemination (**Figure 1**).

This review aims to collate and compare studies that have evaluated biofilm dispersal and eradication combination treatments against established biofilms from clinically relevant pathogens. Important details of the studies cited in this review have been summarised in a comprehensive table (**Table S1**), where the efficacy of the standalone treatments (biofilm dispersal agent or antibiotic) was compared to the efficacy of the combination treatment as reported within each study. For studies where the combination efficacy was greater than the sum of the standalone strategies, these have been included in a summary table (**Table 1**) showcasing the most promising anti-biofilm combination treatments currently under intense study.

**Table 1:** List of promising combination treatments against bacterial biofilms currently in development.

Dispersal Agent	Antibiotic	Tested Species	Treatment Efficacy (Reduction over untreated biofilm)			*In vivo*	Ref
			Combination	Dispersal Agent	Antibiotic		
**Quorum Sensing Inhibitors (QSIs)**							
Baicalin hydrate, cinnamaldehyde, hamamelitannin	Tobramycin, clindamycin, vancomycin	*P. aeruginosa*	**68-90%^1^**	<1%	45%		(Brackman et al., 2011)*
Hamamelitannin analogue 38	Vancomycin, cephalexin	*S. aureus*	**5.75-log^2^**	≤1-log	3.75-log	✓	(Vermote et al., 2016)*
Cyclodextrin– Hamamelitannin	Vancomycin	*S. aureus*	**5.5-log^2^**	1-log	3.5-log		(Brackman et al., 2016)*
C11	Ciprofloxacin, tobramycin, colistin	*P. aeruginosa*	**4-6-log^3^**	<1-2-log	1-2-log		(Furiga et al., 2015)*
3-amino-7-chloro-2-nonylquinazolin-4(3H)-one (ACNQ)	Ciprofloxacin	*P. aeruginosa*	**80%^4^**	10%	50%		(Singh et al., 2019)*
FS10	Tigecycline	*S. aureus*	**5.75-6-log^2^**	1.5-1.75-log	3.5-4-log	✓	(Simonetti et al., 2016)*
FS8	Tigecycline	*S. aureus*	**5-log^2^**	2-log	2-log	✓	(Simonetti et al., 2013)*
4-dimethylaminocinnamic acid (DCA) and 4-methoxycinnamic acid (MCA)	Tobramycin	*C. violaceum*	**>5-log^2^**	<1-log	≤1-log		(Cheng et al., 2020)
**Nitric Oxide and Nitroxides**							
NO (diazeniumdiolate nanoparticles)	Gentamicin	*P. aeruginosa*	**90%^4^**	≤30%	≤30%		(Nguyen et al., 2016)
NO (diethylamin-cephalosporin-30diazeniumdiolate)	Tobramycin	*P. aeruginosa*	**65%^5^**	50%	<1%		(Soren et al., 2020)
nitroxide 4-carboxy-2,2,6,6tetramethylpiperidine 1-oxyl (CTEMPO)	Ciprofloxacin	*P. aeruginosa*, EHEC	**87-99.3%^6^**	60-71%	<1%		(Reffuveille et al., 2015)
CTMIO	Ciprofloxacin	*S. aureus*	**1024 µM^7^**	>2048 µM	4096 µM		(Verderosa et al., 2019a)*
ciprofloxacin-CTMIO hybrid	N/A^11^		**64 µM^7^**	N/A	N/A	
CTEMPO	Ciprofloxacin	UPEC	**≤800 µM^7^**	>1000 µM	≤800 µM		(Verderosa et al., 2019b)*
Dinitroxide-ciprofloxacin hybrid (CDN11)	N/A		**≤400 µM^7^**	N/A	N/A	
**Antimicrobial Peptides (AMPs)**							
G10KHc	Tobramycin	*P. aeruginosa*	**4-log^2^**	<1-log	<1-log		(Eckert et al., 2006)
LFchimera	Doxycycline	*A. actinomycetemcomitans*	**87%^8^**	6%	3%		(Lachica et al., 2019)
Temporin A (TEMP-A), citropin 1.1 (CIT-1.1) and tachyplesin I linear analogue (TP-1-L)	Colistin	*P. aeruginosa*	**6-log^2^**	≤1-log	1-2-log		(Jorge et al., 2017)*
Melimine, Mel4	Ciprofloxacin	*P. aeruginosa* ^9^	**84-90%^5^**	<1%	65%		(Yasir et al., 2020)*
Melittin (hydrogel)	Tobramycin	*P. aeruginosa*	**4.2-fold^10^**	no change	1.8-fold	✓	(Maiden et al., 2019)
AMP38	Imipenem	*P. aeruginosa*	**62.5 µg/mL^7^**	>500 µg/mL	>500 µg/mL		(Rudilla et al., 2016)*
**Repurposed Drugs**							
Ambroxol	Vancomycin	*S. epidermidis*	**7-log^2^**	<1 log	~3-log	✓	(Zhang et al., 2015)

^1^% reduction in total biofilm colony forming units (CFU).

^2^log reduction in CFU/mL.

^3^log reduction in biofilm CFU/cm^2^.

^4^% reduction in viable biofilm bacteria (CFU).

^5^% reduction in total biofilm biomass.

^6^% Biofilm eradication (CFU).

^7^Minimum Biofilm Eradication Concentration (MBEC).

^8^% reduction biofilm CFU/cm^2^.

^9^against ciprofloxacin-sensitive isolates.

^10^fold reduction in biofilm bioluminescence.

^11^N/A denotes hybrid compound testing, hybrids already contained the antibiotic and were not tested in combination with additional antibiotics.

*Low cytotoxicity to human cell lines or in tested in vivo model.

All studies investigating biofilm co-treatments cited in this review were compiled into a table comparing the efficacy of the dispersal agent, antibiotic alone, and combination treatment (**Table S1**). Where co-treatment was more effective than the sum of the standalone treatments, the combination treatment was deemed promising, and these studies are summarized here under different dispersal agent groups (QSIs, NO/Nitroxides, AMPs, Repurposed Drugs). Other study details provided include the name of the dispersal agent(s) and antibiotic(s), bacterial species tested, and if the combination treatment was tested against biofilm infection in vivo. Efficacy measures for each study are different (as marked in the combination column and relevant footnote); efficacy measures reported are in relation to untreated biofilm controls in all studies.

## Cell-Signalling Disrupters

### Quorum Sensing Inhibitors (QSIs)

Quorum sensing (QS) is a bacterial communication system which allows neighbouring cells to send and receive signal molecules, called autoinducers, in a density-dependent manner. QS has been shown to play a pivotal role in biofilm regulation for many species (Brackman and Coenye, 2015). Targeting QS with inhibitors (QSIs) has been a significant innovation in the antibiofilm field, even though the role of QSIs in biofilm formation and dispersal is not always fully understood for different Gram-positive and Gram-negative bacteria (Brackman and Coenye, 2015). QS inhibition can occur by inhibiting autoinducer synthesis, degradation of signalling molecules, interfering with signal binding, and inhibition of the signal transduction cascade, which results in dysregulated biofilm signalling and subsequently, dispersal or inhibition of the biofilm (Brackman and Coenye, 2015; Jiang et al., 2019). Most QSIs are derived from proteins (autoinducers, transcription factors and regulators) that mediate QS in the bacterial target, acting as competitive inhibitors of these systems.

**Figure 2:**
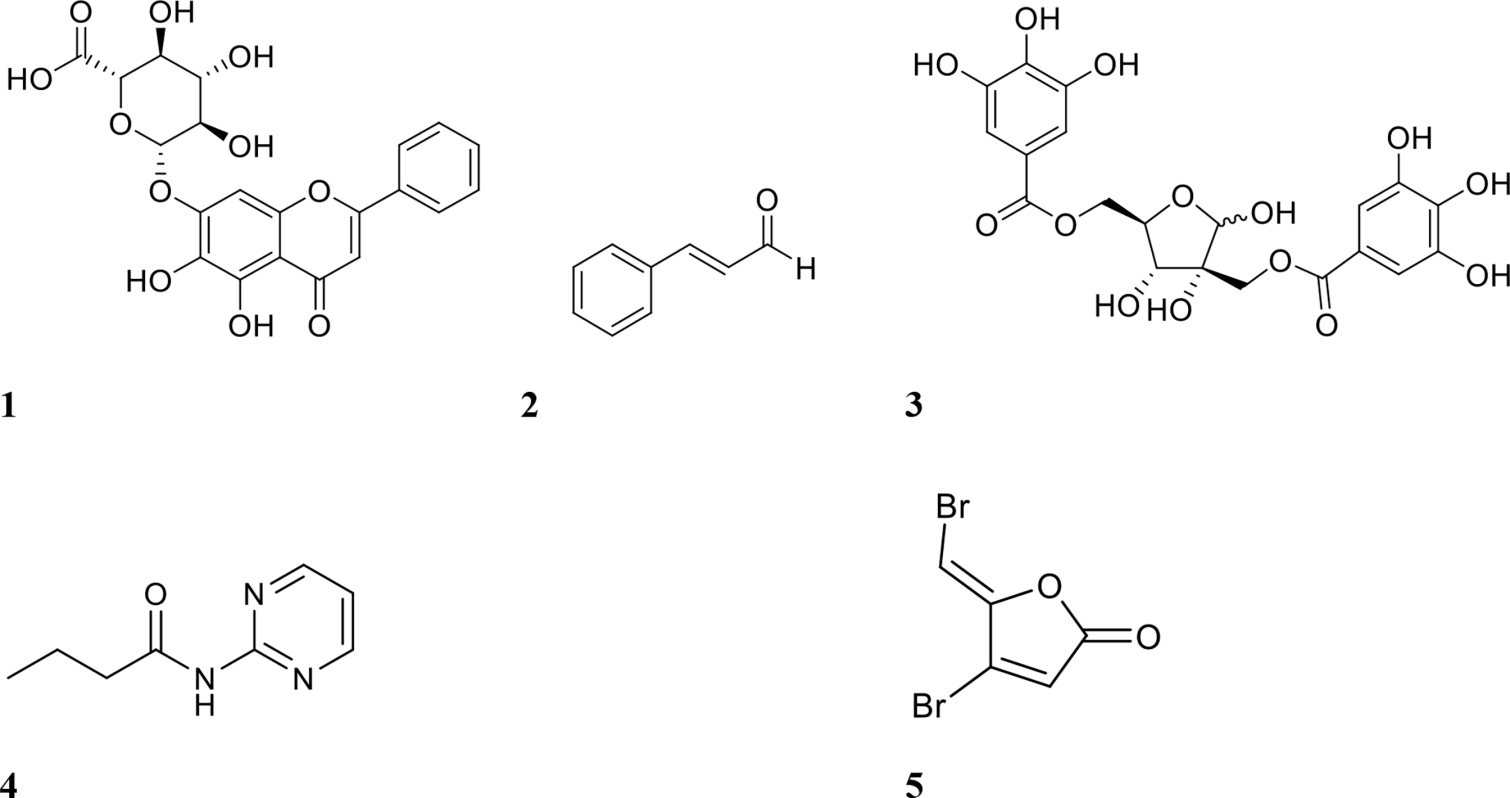
Chemical structures of QSIs 1 baicalin, 2 cinnamaldehyde, 3 hamamelitannin, 4 *N*- (2-pyrimidyl)butanamide (C11), and 5 furanone C-30.

Several studies have been conducted using QSIs in combination with antibiotics to either inhibit and/or eradicate biofilms. One of the earliest studies, conducted by Brackman et al., focused on the efficacies of baicalin hydrate, cinnamaldehyde, and hamamelitannin (structures shown in **Figure 2**) in combination with the antibiotics tobramycin, clindamycin, and vancomycin (Brackman et al., 2011). These compounds were tested against established (24-hour) *Pseudomonas aeruginosa* and *Staphylococcus aureus* biofilms in Mueller-Hinton agar (Brackman et al., 2011). Initial *in vitro* testing showed that individual treatment with inhibitor or antibiotic alone was largely ineffective (<10% reduction in biofilm bacterial numbers) against *P. aeruginosa* ATCC 9027 and *S. aureus* CS1 and Mu50. Tobramycin alone reduced only *P. aeruginosa* PAO1 biofilm bacterial numbers by 45% (Brackman et al., 2011). When biofilms were treated with a combination of QSI and antibiotic, viable bacteria for all strains showed a 68-90% reduction (**Table 1**), except *S. aureus* Mu50, which were only reduced by 6% when treated with clindamycin and a QSI (Brackman et al., 2011). This strain has been shown to be resistant to clindamycin planktonically, which may explain reduced efficacy also against its biofilms (Cui et al., 2009).

These QSI-antibiotic combinations were also evaluated *in vivo* against *Burkholderia cenocepacia* and *Burkholderia multivorans*, in a *Caenorhabditis elegans* and *Galleria mellonella* survival model and in a mouse lung infection model (Brackman et al., 2011). QSIs alone exacted a strain-dependent effect on the survival of *G. mellonella*, with a minimal protective effect observed against the *Burkholderia* spp. (0-30% survival) but very high protection in larvae infected with *S. aureus* (70-100% survival) (Brackman et al., 2011). In *C. elegans*, none of the QSIs alone exhibited any protective effect (<10% survival) (Brackman et al., 2011). Standalone antibiotic treatment in *C. elegans* showed increased survival compared to the inhibitor only, and this was also observed for *G. mellonella* (Brackman et al., 2011). Combination treatment exhibited the most protective effect, with most combinations resulting in 80-100% survival when infected with either *P. aeruginosa* and *S. aureus*. These outcomes were further confirmed in the mouse model, which showed that the combination of tobramycin (30 mg/kg) and baicalin hydrate (2 mg/kg) reduced pulmonary bacterial numbers by 99.9% (Brackman et al., 2011).

The same authors extended their investigation of hamamelitannin, by testing it in combination with several antibiotics (vancomycin, cefazolin, cefalonium, cephalexin, cefoxitin, daptomycin, linezolid, tobramycin, fusidic acid) against established *S. aureus* biofilms (Brackman et al., 2016). Here they measured the efficacy of single treatment (antibiotic only) and compared it to combination treatment (antibiotic and hamamelitannin). For all antibiotics, combination treatment was at least equally as effective but, in most cases, resulted in greatly increased efficacy (40-70% additional biofilm eradication compared to antibiotic treatment alone) (Brackman et al., 2016). The most effective combinations were hamamelitannin with cefazolin, cefoxitin, tobramycin or fusidic acid, which resulted in ≥90% eradication of the biofilm (Brackman et al., 2016). Similarly, Vermote et al. derived a hamamelitannin analogue (compound-38) that was reported to have a 20-fold lower median minimum bactericidal (MBC_50_) value against *S. aureus* Mu50 planktonic cells (Vermote et al., 2016). Mirroring the initial hamamelitannin study (Brackman et al., 2011), all combinations were at least as, or more effective than their standalone counterpart (Vermote et al., 2016). The most effective combinations were compound-38 with vancomycin, cefazolin, daptomycin or tobramycin (≥90% eradication, **Table 1**) (Vermote et al., 2016). Combined, these studies suggest that hamamelitannin is most effective against biofilm bacteria when administered with the antibiotics cefazolin and tobramycin (Brackman et al., 2016; Vermote et al., 2016).

In a different study, Brackman et al. incorporated hamamelitannin into a delivery system to improve its efficacy against *S. aureus* biofilms (Brackman et al., 2016). They achieved this by incorporating the inhibitor into a cyclodextrin complex which could release the QSI and an antibiotic at a controlled rate (Brackman et al., 2016). Utilising these complexes as a delivery system increased efficacy *in vitro*, and the use of the delivery system with hamamelitannin alone reduced biofilm CFU by 1 log (Brackman et al., 2016). However, when the system was used in combination with both vancomycin and hamamelitannin, biofilm CFUs were reduced by 5.5 logs (**Table 1**) (Brackman et al., 2016). Hamamelitannin thus appears to demonstrate a broad-spectrum of biofilm-eradication potentiating activity when administered as a co-treatment with antibiotics. Furthermore, it synergised well with antibiotics from different classes. These properties make hamamelitannin one of the more promising QSIs reviewed here, warranting further exploration into its synergistic capabilities. Given that hamamelitannin has already been tested *in vivo* in mice, this warrants further investigation into its potential toxicity and side effects, before moving into clinical development. As successful analogues with similar efficacies have also been developed (Vermote et al., 2016), this dispersal agent has potential to be modified chemically as well.

Other promising QSIs have also been tested in combination with antibiotics against biofilms. Some of these include furanone C-30 and C11 (structures shown in **Figure 2**), ajoene (garlic extract), 3-amino-7-chloro-2-nonylquinazolin-4(3H)-one (ACNQ) (PqsR receptor inhibitor), derivatives of cinnamic acid, horseradish extract and alkylquinolone-based inhibitors. Christensen et al. investigated combinations of tobramycin and the inhibitors furanone C-30, ajoene, and horseradish extract against *P. aeruginosa* biofilm infection *in vivo* using a mouse intraperitoneal implant model (Christensen et al., 2012). Tobramycin alone failed to reduce bacterial numbers by more than 1 log, but combination treatment reduced bacteria by up to 3 logs (Christensen et al., 2012). Treatment with inhibitor alone however, also showed similar efficacy, suggesting that the interaction between the two compounds was additive rather than synergistic. A derivative of a signalling molecule in *P. aeruginosa* QS, C11, was also tested in combination with the antibiotics ciprofloxacin, tobramycin, ceftazidime, and colistin against forming *P. aeruginosa* biofilms (Furiga et al., 2015). Treatment with individual agents reduced biofilm surface area (CFU/cm^2^) by 1-2 logs, while combination treatment with antibiotics and C11 resulted in a 4-6-log reduction (**Table 1**) (Furiga et al., 2015). The only combination which was not synergistic was ceftazidime and C11, reducing biofilm surface area only by 1-2 logs (Furiga et al., 2015).

Building upon this work, Singh et al. used an inhibitor similar to C11 which also targets QS in *Pseudomonas* spp (Singh et al., 2019). Using engineered polymeric nanoparticles as a delivery system, they co-delivered the inhibitor ACNQ (4 µg/mL) and ciprofloxacin (60 µg/mL) to treat established *P. aeruginosa* biofilms (Singh et al., 2019). Individual treatments with the inhibitor and antibiotic were somewhat effective (10% and 50% eradication, respectively), however, combination treatment significantly enhanced eradication of established biofilms (80% eradication; **Table 1**) (Singh et al., 2019). Using an alternative delivery system, Ho et al. tested the alkylquinolone QSI [1] (20 µM) in combination with tobramycin (25 µg/mL) to treat *P. aeruginosa* biofilms (24-hours) (Ho et al., 2020). Here they employed a squalenyl hydrogen sulfate nanoparticle delivery system to deliver both compounds at the same time (Ho et al., 2020). Treatment with the antibiotic alone resulted in a 4-5-log reduction in biofilm CFU compared to the untreated control, regardless of the mode of delivery (Ho et al., 2020). Combination treatment with the delivery system achieved complete eradication (>6-log reduction), whereas combination treatment without the delivery system resulted in 3-4-log less eradication (Ho et al., 2020). Thus, the design of the delivery system is important for combination treatment with QSIs. The dual nature of a combination treatment suggests that for it to be successful, both compounds must be in the same environment at the same time to work effectively, as depicted in **Figure 1B**. As standalone treatments do not need to interact with any other compounds, this may be why the delivery system had no impact on their efficacy, as reported in this study. This should be taken into consideration when designing future co-treatment strategies.

Another studied QSI is baicalin (**Figure 2**), a flavonoid isolated from the roots of *Scutellaria baicalensis* (Chinese skullcap) (Slachmuylders et al., 2018). Using this compound, Slachmuylders et al. examined biofilm eradication in combination with tobramycin, gentamicin, kanamycin or neomycin (Slachmuylders et al., 2018). Tobramycin was the most effective against several strains of *Burkholderia* spp. when combined with baicalin hydrate (75-95% increase in biofilm eradication against 5 out of 9 strains tested, compared to antibiotic treatment alone) (Slachmuylders et al., 2018). Similarly, Luo et al. tested baicalin in combination with levofloxacin, amikacin, and ceftazidime against *P. aeruginosa* biofilms (Luo et al., 2017). Amikacin and baicalin were the most effective combination for biofilm inhibition *in vitro*, while *in vivo* (mouse peritoneal implant model), ceftazidime and baicalin were the most effective combination for reducing bacterial numbers (Luo et al., 2017).

Another series of QS inhibitors shown to be active against *S. aureus* are analogues of the RNA-III inhibiting peptide (RIP) (Cirioni et al., 2013; Simonetti et al., 2013; Simonetti et al., 2016). Mouse studies using the QSIs FS3 and FS8 with daptomycin and tigecycline, respectively, demonstrated additive and synergistic efficacy against *S. aureus* biofilms (Cirioni et al., 2013; Simonetti et al., 2013). In these studies, Cirioni et al. and Simonetti et al. implanted grafts (with or without inhibitor) and injected *S. aureus* into the graft site while the antibiotic was administered intraperitoneally (Cirioni et al., 2013; Simonetti et al., 2013; Simonetti et al., 2016). Treatment with the QSI or antibiotic alone resulted in a 2-3-log reduction in bacterial numbers after 7 days of infection, compared to the untreated control group (Cirioni et al., 2013; Simonetti et al., 2013). Treatment with both compounds resulted in an additive effect for FS3 and daptomycin (4-log reduction) and a synergistic effect for FS8 and tigecycline (5-log reduction, **Table 1**) (Cirioni et al., 2013; Simonetti et al., 2013). Following on from this, FS10 was also tested in combination with tigecycline and showed similar results in the same mouse infection model against both methicillin-susceptible *S. aureus* (MSSA) and MRSA (Simonetti et al., 2016). Groups treated with FS10 alone showed minimal CFU reduction after 7 days post implantation (1-2-log reduction), while groups treated with tigecycline only showed higher log reduction in bacterial numbers (3.5-4 logs, **Table 1**) (Simonetti et al., 2016). Combination treatment was the most efficacious against both MSSA and MRSA, reducing bacterial numbers by 5.5-6 logs (Simonetti et al., 2016). Considering that the inhibitor and antibiotic are administered separately, administration of both at the same site could potentially improve efficacy.

Lastly, cinnamic acid, a metabolite of *Cinnamomum cassia* (Chinese cinnamon), has recently been shown to inhibit quorum sensing in bacteria (Vasconcelos et al., 2018). In a study by Cheng et al., two synthesised cinnamic acid derivatives, 4-dimethylaminocinnamic acid (DCA) and 4-methoxycinnamic acid (MCA) were tested in combination with tobramycin against *Chromobacterium violaceum* biofilms (Cheng et al., 2020). Individual treatments were largely ineffective at eradicating established biofilms (≤1-log reduction in biofilm CFU counts), though combination treatment was highly successful and resulted in a 5-log CFU reduction compared to the untreated control, see **Table 1** (Cheng et al., 2020). It is also noteworthy that the core structures of these compounds are amenable to synthetic modification, which makes them ideal candidates for the development of more potent derivatives. In conjunction with this, additional *in vivo/ex vivo* studies should be conducted to test the toxicity of newly modified inhibitors while also assessing discrepancies between *in vitro vs*. *in vivo* effects.

Overall, quorum sensing inhibitors are a promising class of biofilm disruptors. Most QSI studies to date demonstrate that while they are not effective at eradicating biofilms on their own, when used together with antibiotics they can effectively eradicate established biofilms both *in vitro* and *in vivo*. Continued research into QSIs is sorely needed, as many of the mechanisms inhibited by them are poorly understood (Brackman and Coenye, 2015). Additionally, further manipulation of the structures of these inhibitors would open many avenues of therapeutic development, considering their amenability to synthetic modification.

### Nitric Oxide

Nitric oxide (NO) is a free radical colourless gas (at room temperature), and a well-established signalling molecule in eukaryotic organisms (Barraud et al., 2015). One of the earliest uses of NO as a biofilm dispersal agent was documented by Barraud et al. (2006) and since then its potential as a biofilm dispersal agent has been widely documented. While NO concentrations at the mM range are antibacterial (Barraud et al., 2015), inhalation of NO gas at such high concentrations is also acutely toxic to the respiratory tract of humans (Weinberger et al., 2001). However, NO-mediated biofilm dispersal occurs at concentrations sublethal for bacteria (nM range) (Barraud et al., 2006). Thus, the use of NO as an effective biofilm eradication strategy requires supplementation with an antimicrobial agent. NO signals dispersal of bacterial biofilms by interacting with enzymes that affect intracellular concentrations of bis-(3′-5′)-cyclic dimeric guanosine monophosphate (c-di-GMP) (Williams and Boon, 2019). The molecular mechanism was recently characterised in some species of bacteria, including *P. aeruginosa*, *Nitrosomonas europaea*, and *Shewanella oneidensis* (Hossain and Boon, 2017; Hossain et al., 2017; Nisbett et al., 2019). Interaction of NO with NO-sensitive enzymes stimulates the activity of phosphodiesterases (PDEs), which degrade c-di-GMP and signal biofilm dispersal (McDougald et al., 2011).

**Figure 3:**
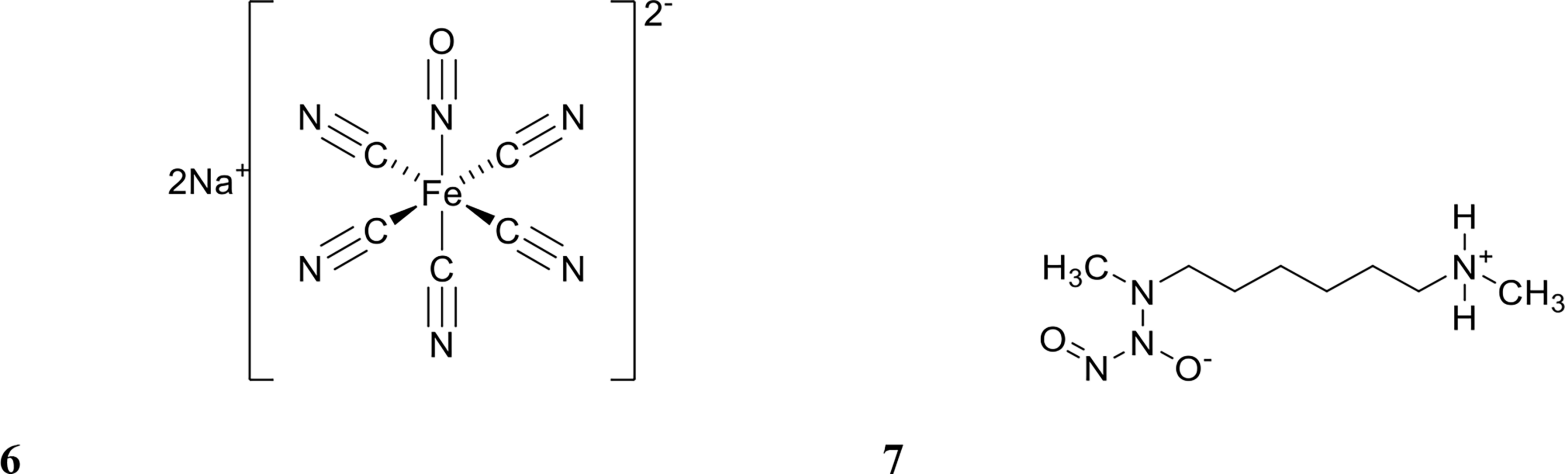
Chemical structures of 6 sodium nitroprusside (SNP) and 7 (Z)-1-[N-Methyl-N-[6-(N-methylammoniohexyl)amino]diazen-1-ium-1,2-diolate (MAHMA NONOate).

Several studies have tested NO for its ability to potentiate antibiotics *in vitro* (Barraud et al., 2009; Nguyen et al., 2016; Liu et al., 2020; Soren et al., 2020). NO is a difficult molecule to work with due to its gaseous form at room temperature and high reactivity (Barraud et al., 2015). Hence, most studies have used NO donors, such as sodium nitroprusside (SNP) and (Z)-1-[N-Methyl-N-[6-(N-methylammoniohexyl)amino]]diazen-1-ium-1,2-diolate (MAHMA NONOate) (Barraud et al., 2009; Barnes et al., 2013; Marvasi et al., 2015) (structure shown in **Figure 3**), which albeit are also known to be inherently unstable molecules (Wang et al., 2002). In order to address this, several NO delivery systems have been reported to release NO in a controlled manner at the target site (Lu et al., 2013; Nguyen et al., 2016; Ren et al., 2016; Liu et al., 2020). Examples of these include diazeniumdiolate (NONOate), micro/nanoparticles, and amphiphilic poly(amidoamine) (PAMAM) dendrimers (Lu et al., 2013; Nguyen et al., 2016; Soren et al., 2020). These systems improve handling and dose mediation, prevent or limit off-target effects, and increase the efficacy of biofilm dispersal by NO while minimising cytotoxicity to the host (Davies et al., 2001; Findlay et al., 2004; Poh et al., 2017; Yang et al., 2018; Verderosa et al., 2019c). In this section, we review studies that have combined NO with antibiotics for either dispersing or eradicating clinically relevant biofilms, while excluding studies which have only tested NO alone *in vitro*.

In one of the earlier *in vitro* studies, Barraud et al. investigated the potentiation of tetracycline by NO against biofilms from a variety of different bacterial species, using the NO-donor sodium nitroprusside (SNP) (Barraud et al., 2009). Pre-treating *Vibrio cholerae* biofilms with NO and then adding tetracycline reduced the biofilm surface area by 90% compared to untreated biofilms (Barraud et al., 2009). NO alone reduced the biofilm surface area by 67%, while tetracycline alone only afforded a 21% biofilm reduction (Barraud et al., 2009). The study also investigated the ability of NO to potentiate chlorine used for water treatment. Similar to tetracycline, NO treatment of *V. cholerae* biofilms followed by chlorine resulted in significant biofilm reduction (85-90%) compared to untreated controls (Barraud et al., 2009). The authors concluded that NO could enhance the activity of tetracycline and chlorine against biofilms when administered sequentially (NO treatment followed by tetracycline/chlorine treatment), however co-treatment with NO and tetracycline was not investigated, nor was the viability of the dispersed cells.

While sequential treatment of biofilms with a dispersal agent followed by an antibiotic is informative and may find use in industrial settings, such as water treatment, this approach would not be suited to the clinical setting. Dispersing a clinical biofilm prior to antimicrobial intervention can lead to systemic bacterial dissemination, which can result in spread of infection, septicaemia, and bacteraemia (Fleming and Rumbaugh, 2018; Müsken et al., 2018). For this reason, subsequent biofilm studies have focused on NO co-treatment with antibiotics.

NO co-administration with antibiotics has been facilitated by delivery systems such as those developed by Soren et al. which use the prodrug diethylamin-cephalosporin-30diazeniumdiolate (DEA-C3D) to investigate the delivery of NO in combination with tobramycin to eradicate *P. aeruginosa* biofilms (Barraud et al., 2012; Soren et al., 2020). DEA-C3D belongs to a class of NO-donor prodrugs known as cephalosporin-30-diazeniumdiolates (C3Ds), which contain the phenacetyl side chain of the first-generation cephalosporin cefaloram and the NO donor diazeniumdiolate (Soren et al., 2020). These compounds deliver NO by local release upon contact with bacterial β-lactamase enzymes (Soren et al., 2020). When used to deliver NO for the treatment of *P. aeruginosa* biofilms, NO alone reduced biofilm biomass by 50%, while treatment with the antibiotic tobramycin alone had no effect on biofilm biomass. However, NO and tobramycin co-delivery reduced biofilm biomass by 65%. While the potentiation of tobramycin was relatively modest, it should be noted that the concentration of antibiotic used in the study was sublethal (Soren et al., 2020), suggesting that efficacy could be likely increased by using tobramycin at higher concentrations. A similar potentiation trend was also observed for colistin. Despite colistin treatment alone being extremely effective against *P. aeruginosa* biofilms (90% biomass reduction), co-treatment with DEA-C3D resulted in almost complete biofilm eradication (98% reduction, **Table 1**) (Soren et al., 2020). The design of this NO delivery strategy is certainly innovative; however, its application is limited to β-lactamase-producing bacteria, and furthermore, it is unfortunate that the antibacterial properties of the delivery system (β-lactam) are lost upon NO release, which was an intentional design feature (Soren et al., 2020). The authors comment that the ultimate goal of this delivery system is to deliver NO to a targeted site, containing β-lactamase-producing bacteria, to reduce tissue damage from NO’s reactivity (Soren et al., 2020). In addition, as the study determined biofilm eradication by visualising remaining cells, dispersed cells were not studied. This makes it difficult to evaluate the efficacy of this treatment in the context of a “disperse and eradicate” strategy, as it is important to determine whether dispersed cells remained viable or were killed.

Another study targeting *P. aeruginosa* biofilms used a diazeniumdiolate nanoparticle delivery system in combination with the antibiotic gentamicin (Nguyen et al., 2016). Here, Nguyen and co-workers designed and produced a polymeric nanoparticle delivery system that simultaneously released NO and gentamicin at a target site by placing a gentamicin-NONOate complex (generated by reacting gentamicin and NO gas) at the core of the nanoparticle (Nguyen et al., 2016). Co-treatment with NO and gentamicin reduced biofilm viability by 90%, whereas treatment with the individual compounds only reduced viability by ≤30%, (**Table 1**) (Nguyen et al., 2016). These results are promising and should the compound prove to be non-toxic to mammalian cells, a property that was not examined, the strategy would certainly merit *in vivo* analysis. Similarly, Liu et al. engineered a NO delivery system using a derivative of a naturally occurring compound, chitosan (Liu et al., 2020). The donor, chitosan-graft-poly(amidoamine) dendrimer (CS-PAMAM), was used to deliver methicillin and NO to methicillin-resistant *Staphylococcus aureus* (MRSA) biofilms (Liu et al., 2020). They observed that despite the strains being methicillin resistant, co-treatment with methicillin increased biofilm eradication a further 10% than with NO treatment alone (Liu et al., 2020). This study is unique as the target pathogen has acquired resistance to the antibiotic yet co-treatment with NO had an effect, which we speculate suggests that NO was likely dispersing cells from the biofilm outer layer and allowing higher levels of methicillin to access and kill underlying biofilm-residing cells.

The above studies have showcased the promising potential of NO as a biofilm dispersal agent with and without antibiotics *in vitro*. However, studies on NO co-treatments of clinical biofilms using *in vivo* models (cystic fibrosis, urinary tract infections, chronic wounds, etc. (Vestby et al., 2020)] are currently lacking. Nevertheless several *in vivo* studies on NO have provide valuable insight into its potential use as an infection treatment and or control strategy (Webert et al., 2000; Miller et al., 2012; Miller et al., 2013).

Inhaled NO has been studied as a standalone treatment for pneumonia in rats and has more recently progressed to phase I human trials (Webert et al., 2000; Miller et al., 2012; Miller et al., 2013; Deppisch et al., 2016). A rat model of *P. aeruginosa* pneumonia was initially used to test the efficacy of inhaled NO (Webert et al., 2000; Miller et al., 2012; Miller et al., 2013). Rats were injected intratracheally with 10^8^ colony forming units (CFU) of *P. aeruginosa* and then monitored over time for any physiological changes and for bacterial carriage in the lungs (Webert et al., 2000). The study successfully demonstrated that NO could reduce the pulmonary bacterial load after 24 hours of NO inhalation at a concentration of 40 ppm (Webert et al., 2000). A follow up study by the same group demonstrated that an even higher concentration of NO (160 ppm) given intermittently was also effective at lowering viable *P. aeruginosa* counts in the lungs of infected rats (Miller et al., 2013). This confirmed previous *in vitro* studies, which had shown that at a concentration of 160ppm, NO is bactericidal (Schairer et al., 2012). The success of these preclinical animal studies paved the way for a small Phase I study on 10 healthy adult volunteers, which confirmed the same dosing regimen of 160 ppm for 30 minutes was safe (Miller et al., 2012). A follow-up pilot clinical study treated eight cystic fibrosis patients suffering from long term (>6 months) bacterial and/or fungal lung infection(s) with gaseous NO for 30 minutes, three times daily, at a concentration of 160 ppm for two periods of five days over two weeks (Deppisch et al., 2016). Patients were followed over the course of seven months and post-NO treatment were all found to have an average log-reduction of 3.6 and 3.0, respectively, of bacteria and fungi (regardless of species) in their sputum samples (Deppisch et al., 2016). In addition to this, ESBL-producing *Escherichia coli* or *Aspergillus* spp. biofilms were undetectable after treatment. This study is one of the first of its kind and is unique from other NO dispersal studies in that it did not require the use of a NO delivery system. Additionally, these studies demonstrate the safety of NO administration even at bactericidal concentrations, which are higher than the concentrations shown to be required to disperse bacterial biofilms *in vitro*. Future clinical studies will greatly enhance the development of new respiratory therapeutics with potential benefits for other respiratory diseases.

### Nitroxides

The development of antibacterial and antibiofilm therapies based on NO is often limited by its high reactivity, instability, and inherent human toxicity at high concentrations (Barraud et al., 2015; Verderosa et al., 2019c). Thus, alternatives that mimic the behaviour of NO, but lack its limitations, have recently been explored (de la Fuente-Núñez et al., 2013; O'Loughlin et al., 2013; Rajasekaran et al., 2019).

**Figure 4:**
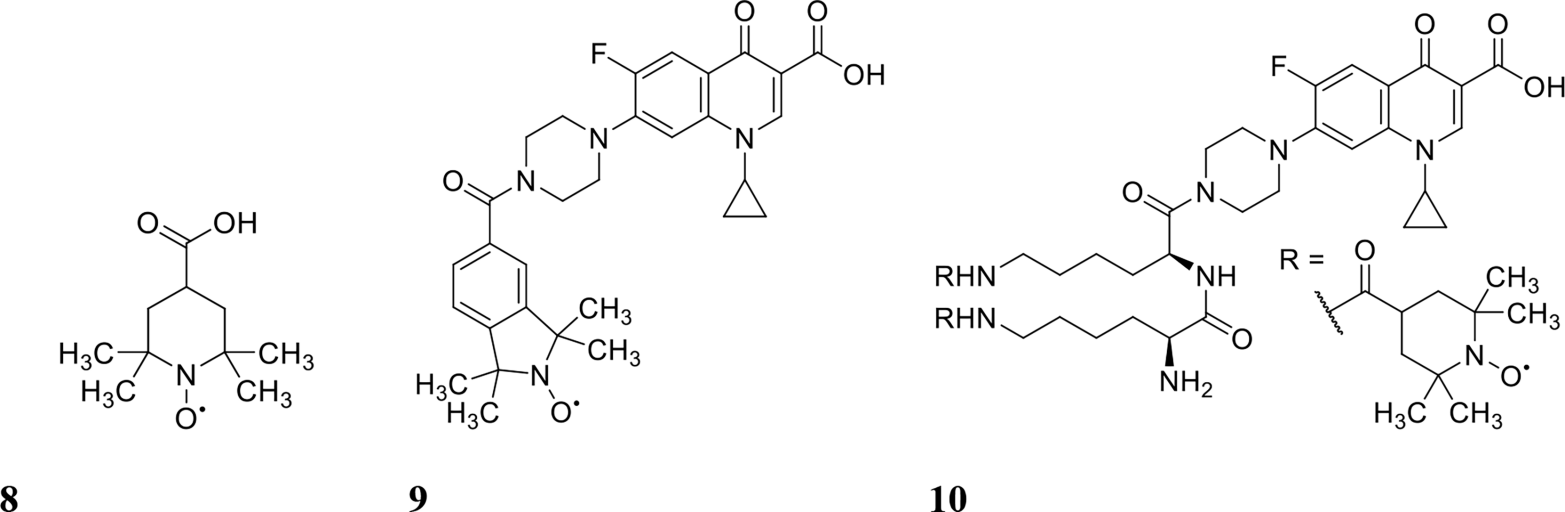
Chemical structures of 8 4-carboxy-2,2,6,6tetramethylpiperidine 1-oxyl (CTEMPO), 9 ciprofloxacin-nitroxide hybrid-27 and 10 dinitroxide-ciprofloxacin hybrid 11 (CDN-11).

Nitroxides are structurally similar to NO as both contain an unpaired electron delocalised over the nitrogen-oxygen bond. However, unlike NO, nitroxides are generally air-stable crystalline solids at room temperature, which makes their handling and delivery far more convenient than NO. Furthermore, nitroxides have been recently shown to exhibit similar antibiofilm properties to NO *in vitro* (de la Fuente-Núñez et al., 2013; Verderosa et al., 2016). The antibiofilm properties of nitroxides were first explored by de la Fuente Núñez et al., who showed that nitroxides not only mimicked the biofilm dispersal activity of NO but also prevented biofilm formation *in vitro* (de la Fuente-Núñez et al., 2013). In a follow up study by the same group, nitroxides were shown to potentiate the action of ciprofloxacin against both *P. aeruginosa* and *E. coli* biofilms (Reffuveille et al., 2015). Here, they investigated the ability of the nitroxide 4-carboxy-2,2,6,6tetramethylpiperidine 1-oxyl (CTEMPO) (structure shown in **Figure 4**) to potentiate the activity of ciprofloxacin when administered as a co-treatment (Reffuveille et al., 2015). CTEMPO (20 µM) alone dispersed *P. aeruginosa* and enterohemorrhagic *E. coli* (EHEC) biofilms resulting in significantly reduced biomass (60% and 71%, respectively, **Table 1**) (Reffuveille et al., 2015). At the same concentration, CTEMPO also potentiated ciprofloxacin activity against *P. aeruginosa* and EHEC biofilms, eradicating 99.3% and 93%, respectively (Reffuveille et al., 2015). This occurred at a ciprofloxacin concentration of just 320 ng/mL for *P. aeruginosa* and 20 ng/mL for EHEC when ciprofloxacin treatment alone had no or minimal effect on biofilm biovolume (Reffuveille et al., 2015). Dispersed cells from CTEMPO treated *P. aeruginosa* biofilms were also collected and quantified at 3-, 6- and 24-hours post treatment. Between the 3 and 6 hour treatment timepoints, there was minimal change in bacterial numbers, however after 24 hours, there was a minimum 5-fold change in dispersed bacterial counts (Reffuveille et al., 2015). This suggests that after 24 hours of CTEMPO treatment, the biofilm has been sufficiently dispersed that many cells are becoming dislodged and flowing out of the system.

Verderosa et al. built upon this work by synthetically linking the nitroxide to the ciprofloxacin moiety to create a variety of ciprofloxacin-nitroxide hybrids (Verderosa et al., 2016; Verderosa et al., 2017; Verderosa et al., 2019a; Verderosa et al., 2019b). These hybrid compounds were effective in dispersing and eradicating both Gram-negative (*P. aeruginosa*, uropathogenic *E. coli* (UPEC)) as well as Gram-positive (*S. aureus*) biofilms *in vitro* (Verderosa et al., 2017; Verderosa et al., 2019a; Verderosa et al., 2019b). *P. aeruginosa* biofilms were 94% eradicated with ciprofloxacin-nitroxide hybrid-27 (structure shown in **Figure 4**) treatment at 20 µM (Verderosa et al., 2017). Treatment with the hybrid was on par with the synergy reported previously against *P. aeruginosa* biofilms using CTEMPO and ciprofloxacin co-treatment (Reffuveille et al., 2015). However, hybrid compounds provide some intrinsic advantages over co-treatments, such as *i*) more favourable pharmacokinetic and pharmacodynamic properties and *ii*) insurance that the dual action of dispersal and eradication are contained in the one compound, which is especially important for *in vivo* studies (Verderosa et al., 2017; Fleming and Rumbaugh, 2018). In a follow-up study by Verderosa et al., co-treatment and treatment with hybrid compounds was tested against *S. aureus* biofilms (Verderosa et al., 2019a). Co-treatment with nitroxide (8 µM for CTEMPO) and ciprofloxacin (256 µM) was effective at eradicating biofilms, affording a 16-fold improvement (**Table 1**) in the minimum biofilm eradication concentration (MBEC) (99.9% eradication) compared to ciprofloxacin treatment alone (Verderosa et al., 2019a). Interestingly, treatment with the ciprofloxacin-nitroxide hybrid-27 at 64 µM was 4-fold more potent than co-treatment and 64-fold more potent than ciprofloxacin alone, suggesting that hybrid compounds (dispersal agent linked to antibiotic) may provide distinct advantages over co-treatments against some species (Verderosa et al., 2019a). In a subsequent study, the authors developed a synthetic strategy for altering the ratio of nitroxide to antibiotic producing a dinitroxide-ciprofloxacin hybrid (CDN-11, structure shown in **Figure 4**) with potent activity against UPEC biofilms *in vitro* (Verderosa et al., 2019b). CDN-11 was effective in eradicating biofilm residing UPEC, with a 99% reduction in bacterial numbers (*vs*. untreated group) at 12.5 µM (**Table 1**) (Verderosa et al., 2019b). The use of nitroxides in the eradication of biofilms is certainly encouraging, especially considering how well they can potentiate the activity of ciprofloxacin (either as a co-treatment or in hybrid compounds). However, their ability to potentiate the activity of other classes of antibiotics remains to be explored as does their antibiofilm activity *in vivo*. The lack of human cell toxicity reported for nitroxides and nitroxide functionalised antibiotics (Sadowska-Bartosz et al., 2015; Verderosa et al., 2017) however supports their further development as antibiofilm antimicrobials.

Clearly, NO and nitroxides are effective biofilm dispersal agents and based on *in vitro* and *in vivo* studies, they both hold genuine promise as treatment strategies for biofilm related infections. As co-treatment agents they have also showed synergy with several antibiotics and proved to be effective at eradicating established biofilms. While the combined use of NO, nitroxides and antibiotics is still in early preclinical testing, its development towards clinical applications is likely to progress. Continued assessment against other biofilm-forming organisms (*in vitro* and *in vivo*) is needed to ascertain how broad-spectrum these combinations can be. NO has already been tested against several well established biofilm-forming species, including the fungal pathogen *Candida albicans*, and proven efficacious for biofilm dispersal (Barraud et al., 2009). Further work expanding the testing of such combination treatments towards several other species would aid both their development as therapeutics and understanding of the mechanism behind NO’s biofilm dispersing ability, which is likely to differ between microbes.

## Peptides/Membrane Disrupters

### Antimicrobial Peptides

Antimicrobial peptides (AMPs) are small cationic and amphipathic molecules of 12-50 amino acids that have the ability to disrupt bacterial membranes (Grassi et al., 2017). AMPs exhibit a broad-spectrum of activity and have a high potential to target metabolically dormant cells in biofilms (Grassi et al., 2017). AMPs are often studied in combination with antibiotics and other antibiofilm compounds, and many of these studies have been briefly described by Grassi et al. in 2017 (Grassi et al., 2017). Here, we report the efficacy afforded through combination treatment compared to standalone and also include studies that have been published since then.

An early study on AMP-antibiotic combinations assessed the synergistic activity of the synthetic AMP G10KHc (amino acid sequence shown in **Table 2**) in combination with tobramycin for the eradication of *P. aeruginosa* biofilms (Eckert et al., 2006). Treatment with G10KHc or tobramycin alone (each at 100 µg/mL) for 4 and 24 hours had little effect on established biofilms (Eckert et al., 2006). However, when used together at the same concentrations, a 4-log reduction in bacterial numbers (**Table 1**) was observed after 4 hours of co-treatment, with no viable CFU recovered from the biofilms after 24 hours (Eckert et al., 2006). The same group further investigated how G10KHc potentiated tobramycin activity by staining cells with propidium iodide (PI) and observing whether the dye was present in treated cells, as it cannot enter cells with intact membranes (Krishan, 1975; Eckert et al., 2006). Bacterial cells treated with G10KHc were found to fluoresce, indicating that the peptide disrupted the cell membrane, subsequently allowing tobramycin to enter and kill cells (Eckert et al., 2006).

**Table 2:** Amino acid sequences of antimicrobial peptides (AMPs) with notable antibiofilm activity.

Peptide Name	Type	Sequence
G10KHc	Synthetic	KKHRKHRKHRKHGGSGGSKNLRRIIRKGIHIIKKYG
Nisin-A	Natural	MSTKDFNLDLVSVSKKDSGASPRITSIS LCTPGCKTGALMGCNMKTATCHCSIHVSK
Melittin	Natural	NH_2_-GIGAVLKVLTTGLPALISWIKRKRQQ-CONH_2_
1018	Synthetic	VRLIVAVRIWRR-NH_2_
DJK-6	Synthetic	VQWRRIRVWVIR-CONH_2_

#### Naturally Derived Antimicrobial Peptides

A wide variety of inherently antimicrobial peptides exist in nature serving as innate defence proteins against bacteria and produced by many species. Tachyplesin III, for example, is a short peptide from Southeast Asian horseshoe crabs that is similar in structure to the protegrin peptide family, which is also known for its antimicrobial properties (Minardi et al., 2007). Minardi et al. investigated the synergy between Tachyplesin III and piperacillin-tazobactam to treat *P. aeruginosa* biofilms in a rat ureteral stent model (Minardi et al., 2007). Treatment with either Tachyplesin III (10 mg/L stent coating) or piperacillin-tazobactam (120 mg/kg intraperitoneally) resulted in a 3-log reduction of bacterial numbers compared to untreated controls (Minardi et al., 2007). Administered together however, the combination treatment resulted in a 5-log reduction of bacterial numbers, demonstrating synergistic activity (Minardi et al., 2007). In a subsequent study, the AMP BMAP-28 was used in combination with vancomycin to treat *Enterococcus faecalis* and *S. aureus* biofilms (Orlando et al., 2008). The co-treatment was tested both *in vitro* and *in vivo*, again in the rat stent model (Orlando et al., 2008). *In vitro*, a 4-fold decrease in MBEC value was observed, compared to individual treatments (Orlando et al., 2008). *In vivo*, stent cultures taken 5 days post implantation and urine cultures taken 24 hours post implantation showed a 2-3 log reduction in bacterial numbers for both bacterial species when compared to individual treatment groups, and a 5-log reduction compared to untreated controls (Orlando et al., 2008). Despite the agreement between *in vitro* and *in vivo* results, it is important to note that they are not directly comparable, as the methods for quantifying biofilm eradication were different (MBEC *vs* CFU quantification).

Similar to Tachyplesin III, lactoferrin is another peptide with antibacterial and bacterial anti-adhesion properties, and is commonly found in human blood and secreted fluids (Ammons and Copié, 2013). Wakabayashi et al. showed that treatment of *Porphyromonas gingivalis* biofilms with a combination of lactoferrin and ciprofloxacin was very effective, reducing biofilm biomass by 80% (0.5 mg/mL lactoferrin, 10 µg/mL ciprofloxacin) whereas the compounds alone only reduced biofilm biomass by 50% and 40%, respectively (Wakabayashi et al., 2009). Following on from this work, Lachica et al. tested lactoferrin chimera (LFchimera) against *Aggregatibacter actinomycetemcomitans* biofilms, a major causative agent of periodontitis (Lachica et al., 2019). Treatment with LFchimera alone or in combination with doxycycline was assessed for biofilm surface area. Combination treatment was most effective, reducing surface area by 87% compared to the untreated control, while individual treatments only reduced area by <10%, (**Table 1**) (Lachica et al., 2019).

Another naturally derived AMP, nisin, has also been assessed for antibiotic potentiation against biofilms *in vitro*. Nisin is a 34 amino acid peptide that originates from *Lactococcus lactis* and is commonly used as a food preservative (amino acid sequence shown in **Table 2**) (Tong et al., 2014) as it has been shown to have strong antimicrobial activity against many Gram-positive bacteria (Tong et al., 2014). Tong et al. investigated the synergy between nisin and several antibiotics for the eradication of *E. faecalis* biofilms *in vitro* (Tong et al., 2014). All nisin-antibiotic combinations were more effective than individual treatments, except for sulphapyridine, metronidazol, and polymyxin, which demonstrated no improved activity over standalone treatment (Tong et al., 2014). Conversely, for Gram-negative bacteria, Field et al. found that combining nisin with polymyxin and colistin was effective in preventing *P. aeruginosa* biofilm formation (Field et al., 2016). Biofilm formation was only slightly inhibited by the compounds alone at sub-MIC levels, but the combination of nisin (at ¼ MIC) and polymyxin or colistin (at either ½ or ⅕ MIC) completely prevented biofilm growth up to 24 hours (Field et al., 2016). The difference between these two studies suggests that nisin is more synergistic with colistin over polymyxin, despite the antibiotics belonging to the same class. Colistin is a membrane-disrupting antibiotic that serves as a last resort treatment for multidrug resistant infections (Bialvaei and Samadi Kafil, 2015). Synergy between AMPs and colistin was also investigated by Jorge et al, using the AMPs temporin A (TEMP-A), citropin 1.1 (CIT-1.1) and tachyplesin I linear analogue (TP-1-L). AMPs were tested individually and in combination with colistin against *P. aeruginosa* and *S. aureus* biofilms (Jorge et al., 2017). All AMP-antibiotic combinations were effective in inhibiting biofilm formation by at least 2 log (CFU/cm^2^) (**Table 1**) (Jorge et al., 2017). The most effective combination was colistin with CIT-1.1, which inhibited formation of *P. aeruginosa* (PAO1) biofilms by 7.7 logs (Jorge et al., 2017). All combinations were also tested for eradication activity against 24-hour established biofilms with only colistin and TP-1-L reported to completely eradicate (6-log reduction in bacterial numbers) *P. aeruginosa* biofilms (Jorge et al., 2017). All other combinations and individual treatments only reduced bacterial numbers by 2 logs or less (Jorge et al., 2017), which suggests that these colistin-AMP combinations are more useful for inhibiting rather than eradicating biofilms. These findings were supported by Mataraci et al., who tested several AMPs (indolicidin, CAMA (cecropin (1-7)–melittin A (2-9) amide), and nisin with multiple antibiotics (daptomycin, linezolid, teicoplanin, ciprofloxacin, and azithromycin) to prevent the formation of methicillin-resistant *S. aureus* biofilms *in vitro* (Mataraci and Dosler, 2012). All combination treatments (AMP/AMP, AMP/antibiotic and antibiotic/antibiotic) were equally effective at preventing biofilm formation (Mataraci and Dosler, 2012). These findings suggest that biofilms are more susceptible to combination treatment during early development stages. This may be due to lack or reduced biofilm features known to contribute to intrinsic resistance [i.e. a matrix of extracellular polymeric substances (EPS) and persister cells (Verderosa et al., 2019c)], allowing access of AMPs and antibiotics to actively growing biofilm cells.

**Figure 5:**
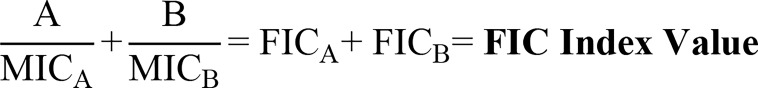
Fractional inhibitory concentration (FIC) index formula. The equation used to calculate synergy, indifference, or antagonism between two compounds (Hall et al., 1983). “A” refers to the MIC value of compound A in combination with compound B, where “MIC_A_” refers to the MIC of compound A alone. “B” refers to the MIC value of compound B in combination with compound A, where “MIC_B_” refers to the MIC of compound B alone. These values added together output the FIC index value, where < 0.5 indicates synergy, 0.5-4 indifference, and > 4 antagonism.

Kalsy et al. investigated antibiotic combinations with the insect derived peptide cecropin A, which is involved in innate immune defence (Kalsy et al., 2020). Cecropin A was tested for synergy with nalidixic acid against uropathogenic *E. coli* (UPEC) biofilms (Kalsy et al., 2020). Interestingly, cecropin A was highly effective at inhibiting both forming and established biofilms, but combination with nalidixic acid did not improve its eradication activity (Kalsy et al., 2020), supporting the tenet that AMPs in general appear to be more effective at inhibiting biofilm formation over eradication. The fractional inhibitory concentration (FIC) index for cecropin A and nalidixic acid was calculated using the FIC formula (**Figure 5**) (Hall et al., 1983), which was reported <0.5 (indicating synergy), which conflicts with their previous findings (Kalsy et al., 2020). The authors state that UPEC was unlikely to harbour intrinsic resistance to the combination treatment, as its mode of action disrupts the bacterial outer membrane and overcoming this would be genetically and metabolically taxing (Kalsy et al., 2020). They then tested cecropin A and nalidixic acid *in vivo* using a *G. mellonella* (greater wax moth) larvae infection model (Kalsy et al., 2020). Combination treatment of moth larvae was also not protective against UPEC challenge (Kalsy et al., 2020). However, when a protease inhibitor was co-administered, all larvae treated with both cecropin A (50 μg/mL) and nalidixic acid (0.5 ng/mL) survived up to six days post injection with *E. coli* (Kalsy et al., 2020). They proposed that the addition of the protease inhibitor prevented proteolytic degradation of cecropin A *in vivo*, allowing it to have maximal activity in combination with nalidixic acid (Kalsy et al., 2020).

**Figure 6:**
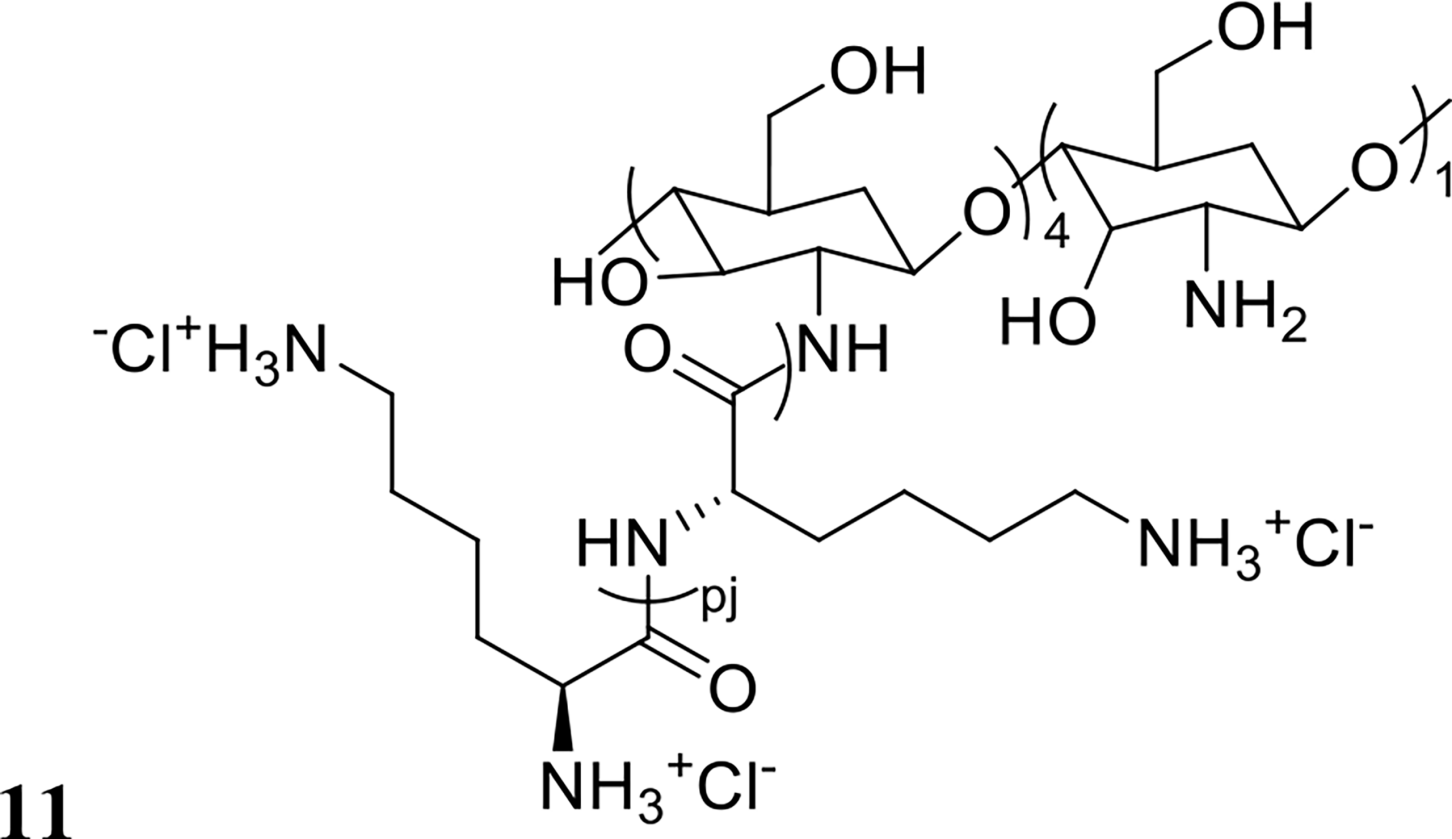
Chemical structure of 11 CSM5-K5.

Thappeta et al., investigated the efficacy of the naturally derived chitosan-based peptide CSM5-K5 (structure shown in **Figure 6**) against *S. aureus* biofilms in combination with the antibiotics oxacillin, meropenem or streptomycin (Thappeta et al., 2020). MBC values were 2-3 fold lower with co-treatment compared to standalone treatment, a finding that was confirmed *in vivo* against *S. aureus*, *E. faecalis* and uropathogenic *E. coli* in a mouse wound excision model (Thappeta et al., 2020). The authors also reported little resistance development after 15 days of serially passaging the bacteria *in vitro* in the presence of CSM5-K5 (Thappeta et al., 2020). Similarly, Yasir et al. reported no resistance development to the AMPs melimine and Mel4 against *P. aeruginosa* biofilms after 30 days of exposure at sub-MIC levels (Yasir et al., 2020). Melimine is a cationic chimera of two naturally occurring peptides: melittin and protamine, and Mel4 is a derivative of melimine with demonstrated antibiofilm activity against *P. aeruginosa* (Yasir et al., 2020). Synergy between these AMPs and the antibiotic ciprofloxacin was assessed by measuring reduction in biomass in established (24-hour) ciprofloxacin resistant or sensitive *P. aeruginosa* biofilms (Yasir et al., 2020). Treatment with each peptide or ciprofloxacin alone at 1x MIC had no effect on ciprofloxacin resistant biofilms, but combination treatment at 1x MIC resulted in 61-66% reduction in biofilm mass (**Table 1**) (Yasir et al., 2020). Synergy was also reported against ciprofloxacin sensitive biofilms, where peptide only treatment at 1x MIC had no effect, and while ciprofloxacin reduced biofilm mass by 65%, combination treatment reduced biofilm mass by 84-90% (Yasir et al., 2020).

Recent advances in clinical treatment include the use of hydrogels as scaffolds for long-term drug release in wounds, to maintain wound sterility and aid healing (Azevedo et al., 2020). Maiden et al. investigated the integration of the AMP melittin (amino acid sequence shown in **Table 2**) in an agarose-based hydrogel in addition to co-treatment with the antibiotic tobramycin. Using an *in vivo* mouse wound model, they reported that tobramycin alone reduced biofilm bioluminescence (used here as a measure of biomass) by 1.8-fold after 4 hours, with melittin having no effect (Maiden et al., 2019). However, incorporating both compounds into the hydrogels reduced biofilm bioluminescence 4.2-fold (**Table 1**) (Maiden et al., 2019). Other studies incorporating hydrogels as delivery systems have also reported increased combination treatment efficacy, potentially due to simultaneous release of compounds, and engineered slow release of the compounds over time (Marvasi et al., 2015; Anjum et al., 2018; Maiden et al., 2019).

These studies on naturally derived AMPs have collectively demonstrated their activity in inhibiting biofilm formation and expansion. However, natural AMPs appear less effective at eradicating established biofilms when administered alone or as part of co-treatment strategies. To overcome this, synthetic AMPs based on natural peptides have recently been engineered and trialled as biofilm eradication agents.

#### Synthetic Antimicrobial Peptides

Synthetic AMPs are peptides that have been synthesised *de novo* and engineered to have antimicrobial properties. As such, they have become a focus for many antibiotic co-treatment studies involving biofilms. Amongst the first synthetic peptides with confirmed antibacterial activities were those engineered by Kovacs et al., who reported their efficacy against Gram-positive and Gram-negative bacteria (Kovacs et al., 1960). Following that, several other synthetic AMPs have been designed and tested against a variety of bacterial pathogens. Synthetic peptide 1018 (amino acid sequence shown in **Table 2**) has been one studied extensively for its activity to potentiate antibiotics against biofilms. Reffuveille et al. initially tested the ability of 1018 to potentiate ciprofloxacin against *P. aeruginosa* biofilms (Reffuveille et al., 2014). Treating biofilms with ciprofloxacin alone at MIC, 10x MIC, and even 100x MIC had little eradication effect. However, combining peptide 1018 with ciprofloxacin, mostly eradicated *P. aeruginosa* biofilms and any remaining cells were small microcolonies of dead cells (Reffuveille et al., 2014). The efficacy of peptide 1018 in combination with other antibiotics was also tested against biofilms from multiple species (*E. coli, S. aureus, Klebsiella pneumoniae, Acinetobacter baumannii*, and *Salmonella enterica*) with combination treatment found always to be most effective when visually comparing biomass reduction and bacterial cell viability (Reffuveille et al., 2014). De la Fuente Núñez et al. also treated *P. aeruginosa* biofilms with the D-enantiomeric peptides DJK-5 and DJK-6 (DJK-6 amino acid sequence shown in **Table 2**) in combination with multiple antibiotics (ceftazidime, ciprofloxacin, imipenem, and tobramycin) (de la Fuente-Núñez et al., 2015). Using confocal microscopy and an *in vivo C. elegans* model, they showed that regardless of the combination, the concentration of antibiotic needed to inhibit biofilm growth was decreased at least 2-fold and in certain cases up to 16-fold with addition of the peptides (de la Fuente-Núñez et al., 2015). Similarly, DJK-6 potentiated meropenem against *K. pneumoniae* biofilms, reducing its MBEC value 16-fold, despite the fact that the tested isolates were carbapenemase producers (Ribeiro et al., 2015). Rudilla et al. also investigated combinations of synthetic peptides with imipenem, reporting that peptide AMP38 decreased by 8-fold the imipenem MBC value for *P. aeruginosa* biofilms (**Table 1**) (Rudilla et al., 2016). Swedan et al. tested AMP WLBU2 in combination with imipenem, tobramycin, amoxicillin-clavulanate or ciprofloxacin against *K. pneumoniae* and *A. baumannii* biofilms, reporting that all combination treatments decreased MBEC values from 6- to 200-fold compared to treatment with WLBU2 alone, the dramatic 200-fold improvement being in combination with ciprofloxacin against *K. pneumoniae* (Swedan et al., 2019). However, when testing cytotoxicity at MBEC values, a significant reduction in eukaryotic cell viability was observed for all tested concentrations of WLBU2 (Swedan et al., 2019). Due to their high cytotoxicity, WLBU2 combination treatment strategies might be more suitable for eradication of biofilms found in the environment e.g. on hospital surfaces or instruments. Alternatively, future efforts could be directed to modifying the chemical structure of WLBU2 to reduce cytotoxicity and facilitate the development of clinically viable antibiofilm agents.

In a more recent study, Pletzer et al. examined optimal combinations of synthetic AMPs with antibiotics to treat ESKAPE pathogen biofilms in an *in vivo* subcutaneous mouse abscess model (Pletzer et al., 2018). Mice infected with fluorescently tagged strains from the ESKAPE group and *E. coli*, inoculated at a dose of ≥10^7^ bacteria to simulate a chronic human wound, were monitored non-invasively for disease progression and treatment efficacy (Pletzer et al., 2018). Treatments were directly injected into the wound and were administered at approximate *in vitro* MIC concentrations (Pletzer et al., 2018). Antibiotic monotherapy even when administered at higher than MIC concentrations was often ineffective against high-density (biofilm) infections, highlighting that *in vitro* MICs are not reliably predictive of *in vivo* efficacy, which the authors also noted (Pletzer et al., 2018). Treatment with AMPs alone reduced wound size and moderately reduced bacterial numbers in the wound (2.2-22-fold decrease) (Pletzer et al., 2018). Notably, the AMP DJK-5 was very effective in combination with antibiotics (ciprofloxacin, gentamicin, meropenem, and vancomycin) in terms of reducing bacterial numbers in all species tested (ESKAPE and *E. coli*) (Pletzer et al., 2018).

While synthetic AMPs have not been studied as extensively as natural AMPs for antibiotic potentiation against biofilms, their promising potential has been clearly demonstrated. Their distinct advantages over naturally occurring AMPs - they can be readily improved *via* chemical manipulation, can be potentially linked to antibiotics, and engineered to be less toxic to mammalian cells- makes this class of antibiofilm agents very promising.

### Amino Acids

Amino acids can exist in nature as a D-isomer or L-isomer, where the orientation of the alpha carbon in the molecule determines its chirality. The L-isomer is most common in ribosomal peptide synthesis, but recently D-amino acids have also been found in mammals as regulators of neurogenesis and brain receptor function, and also components of some bacterial membranes (Cava et al., 2011). These have recently emerged as a class of potential biofilm dispersal agents given their role as regulators of biofilm dispersal (Cava et al., 2011). So far, the mode of action for D-amino acids has only been delineated in *Bacillus subtilis* biofilm dispersal, where the bacteria were reported to release D-leucine, -methionine, -tyrosine, and -tryptophan, which disrupted the amyloid fibers linking biofilm cells together at nanomolar concentrations (Kolodkin-Gal et al., 2010).

**Figure 7:**
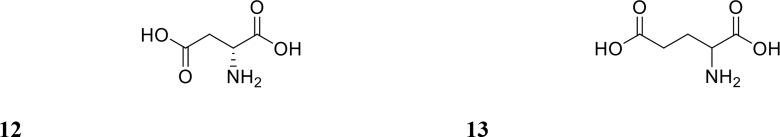
Chemical structures of 12 D-aspartic acid and 13 D-glutamic acid.

While their exact mode of action has yet to be elucidated in other species, a few studies have been conducted using amino acids and antibiotics together as a biofilm treatment strategy. In one of these studies, Sanchez et al. investigated the ability of D-amino acids and antibiotics to inhibit *P. aeruginosa* and *S. aureus* biofilm formation (Sanchez et al., 2014). Here, an equimolar mixture of D-amino acids (methionine, phenylalanine, and tryptophan) in combination with rifampicin were shown to reduce the minimum biofilm inhibition concentration (MBIC) by 4-fold and a >2-log reduction in viable bacteria was reported with treated *S. aureus* biofilms (Sanchez et al., 2014). Similar results were observed for *P. aeruginosa*, with a >2-log reduction in viable bacteria when treated with 64 µg/mL rifampicin, and 5 mM of the D-amino acid mixture (Sanchez et al., 2014). Another study investigating amino acids in combination with antibiotics, conducted by Warraich et al., reported that compared to standalone treatment, lower concentrations of ciprofloxacin and D-amino acids were synergistic (<40 mM amino acids, <90.54 µM ciprofloxacin), and resulted in almost 97% inhibition of biofilm formation and 97.6% dispersal (D-isomer structures shown in **Figure 7**). However, biofilm eradication activity was not tested. The study also investigated the ability of L-amino acids to inhibit biofilm formation, and found that L-isomers of aspartic acid and glutamic acid were equally as effective as the D-isomers at 40mM (Warraich et al., 2020). Only two other studies have reported similar findings with L-amino acids (aspartate and glutamate) (Tong et al., 2014; Yang et al., 2015), challenging the dogma that L-isomers do not have antibiofilm activity. Conflicting evidence reports that L-amino acids can even encourage biofilm formation in some species (Hochbaum et al., 2011; Velmourougane and Prasanna, 2017).

For D-amino acids the evidence is more clear-cut, with several studies reporting some antibiofilm activity (inhibition and dispersal) and in a range of settings, from the industrial to development of drug delivery systems (Si et al., 2014; Wei et al., 2015; Li et al., 2016; Zilm et al., 2017). In these studies, D-amino acids (d-leucine, -methionine, -tyrosine, and -tryptophan) alone and in combination with other compounds effectively inhibited *Desulfovibrio vulgaris* biofilms and polymicrobial biofilms (Si et al., 2014; Wei et al., 2015; Li et al., 2016; Zilm et al., 2017). Li et al. and Si et al. investigated disruption of established *D. vulgaris* biofilms that had been grown on carbon steel coupons (Li et al., 2016) or multi-species aggregates collected from an activated sludge reactor (Si et al., 2014) that had grown for longer than 10 days and up to 6 months (Si et al., 2014; Li et al., 2016). Both studies reported that combinations of D-amino acids with other compounds (hydroxymethyl phosphonium sulfate (THPS) and norspermidine) disrupted biofilm surface attachment (Si et al., 2014; Li et al., 2016). Titanium oxide nanoparticles engineered to release D-amino acids upon stimulation with UV light were also shown effective in dispersing *B. subtilis* biofilms (Wei et al., 2015), and D-amino acids were also found to inhibit *E. faecalis* biofilm formation *in vitro* (Zilm et al., 2017). Some conflicting evidence exists around the capacity of D-amino acids to disperse *P. aeruginosa* and *S. aureus* biofilms (Sarkar and Pires, 2015; Kao et al., 2017). Kao et al. investigated the ability of D-amino acids alanine, leucine, methionine, tryptophan, and tyrosine (10 μM, 1 mM, and 10mM) to inhibit biofilm formation of *P. aeruginosa* strains PAO1 and PA14. Here they concluded that D-amino acids slow biofilm growth but do not prevent its formation (Kao et al., 2017), which Sarkar et al. also report for *S. aureus* biofilms treated with D-tryptophan and D-tyrosine at 1 and 5 mM (Sarkar and Pires, 2015).

Several questions remain unanswered about the antibiofilm properties of amino acids and thus their potential to be employed in future antibiofilm strategies needs more fundamental research. This should aim to resolve the role of both D- and L- isomers in biofilm inhibition and dispersal and explore the specificity of their activity in key biofilm-producing species, such as *P. aeruginosa* and *S. aureus*. Their mode of action is likely to vary between species, and so, future research on this group of potential antibiofilm agents should be prioritized.

## Repurposed Drugs

**Figure 8:**
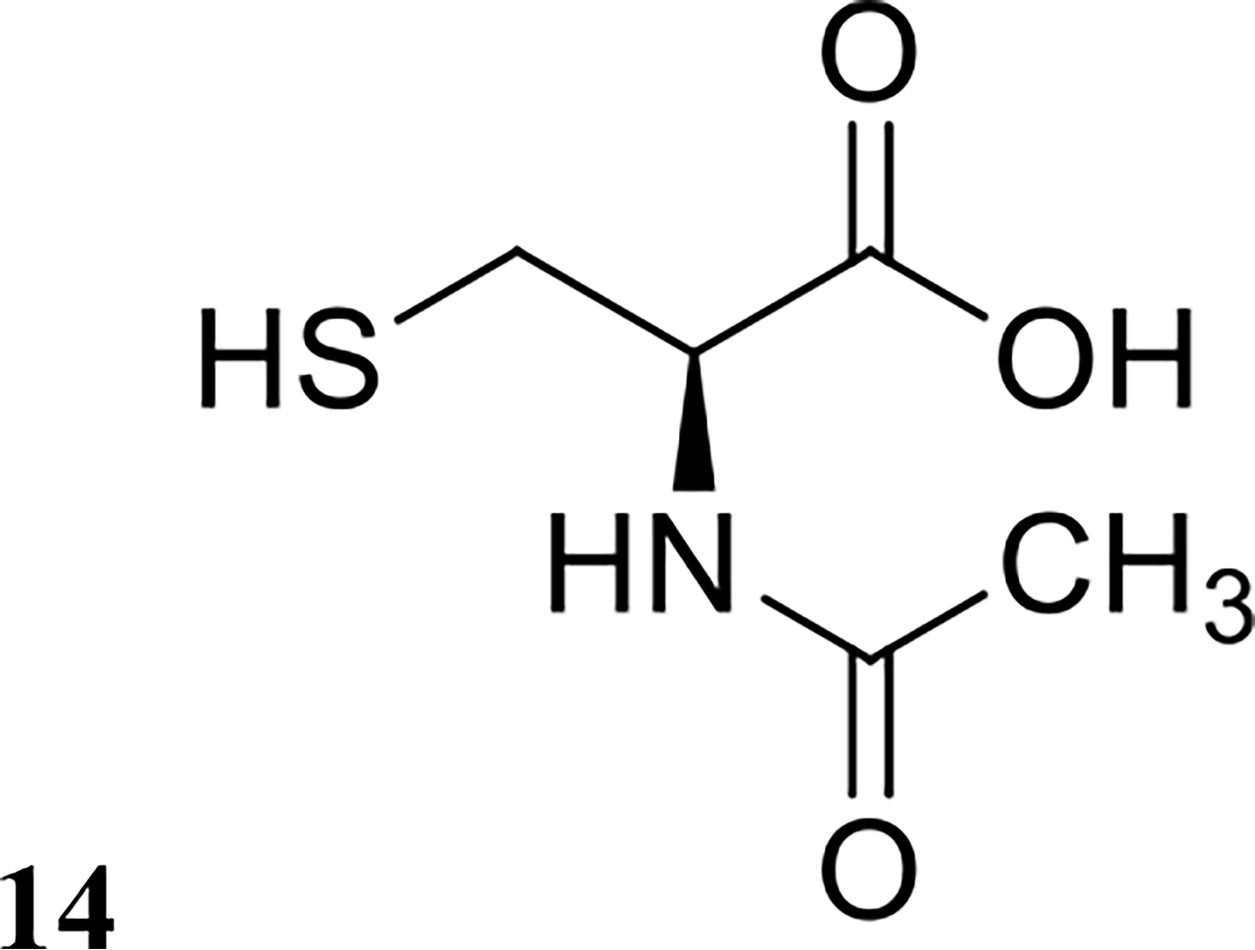
Chemical structure of 14 N-acetyl cysteine.

Drug repurposing is a common strategy used in the field of medicine to maximise the potential of any individual drug for broader clinical applications outside of its original use (Farha and Brown, 2019). A small number of U.S. Food and Drug Administration (FDA)-approved drugs have been investigated against biofilms to date, particularly drugs involved in mucous degradation. *N*-acetyl cysteine (structure shown in **Figure 8**) is a drug commonly prescribed to cystic fibrosis patients to break down mucus in the lung (Samuni et al., 2013). Moon et al. investigated *N*-acetyl cysteine in combination with several antibiotics for dispersal of *Prevotella intermedia* biofilms, a major oral pathogen (Moon et al., 2015). They demonstrated that *N*-acetyl cysteine was highly effective in preventing biofilm growth but was ineffective against pre-formed biofilms *in vitro* (Moon et al., 2015). Zhang et al. also investigated the efficacy of ambroxol (another FDA-approved drug for mucus degradation) in combination with vancomycin to treat *Staphylococcus epidermidis* biofilms *in vitro* and *in vivo* (Zhang et al., 2015). Interestingly, they reported that the combination of ambroxol and vancomycin was highly effective at eradicating mature biofilms in a rabbit intravenous catheter model, reducing catheter bacterial load by an impressive 7 logs compared to the control group (**Table 1**), which were only treated with heparin (Zhang et al., 2015). This result is very promising, and this combination should be further investigated with other bacterial species and other FDA-approved drugs used for mucus degradation.

Another FDA-approved drug with promising antibiofilm activity is auranofin. This gold-containing compound is usually prescribed for the treatment of rheumatoid arthritis, but has since been tested in combination with antibiotics against *S. aureus* and *E. faecalis* biofilms both *in vitro* and *in vivo* (She et al., 2019). Auranofin and the antibiotics fosfomycin, linezolid and chloramphenicol were synergistic at eradicating these biofilms *in vitro*, with compound combinations reducing individual MBEC_50_ values by 2- to over 8-fold (She et al., 2019). *In vivo*, combination treatment was reported to be similarly effective. In a *S. aureus* cutaneous infection mouse model, individual treatment with either auranofin, fosfomycin or linezolid resulted in a <2-log reduction in viable bacterial numbers, while combination treatment with an antibiotic and auranofin resulted in >3-log reduction in *S. aureus* CFU (She et al., 2019). In the same mouse model, however, treatment of *E. faecalis* infection was not reduced by more than 1-log in viable CFU irrespective of the treatment strategy, indicating that *E. faecalis* may have intrinsic resistance to auranofin both planktonically and in sessile form *in vivo*, despite the drug having activity *in vitro* (She et al., 2019).

While the potential use of FDA-approved drugs against biofilms is supported by some impressive results to date, evidence remains sporadic and additional studies are warranted. It would be interesting to investigate other existing mucolytic drugs, as well as approved anticoagulant/antithrombotic and expectorant agents already approved for clinical use.

## Conclusions

The combination of biofilm dispersal agents and antibiotics are an effective treatment strategy for both the prevention and eradication of bacterial biofilms. Many of these combinations have been successfully tested *in vitro*, and some also *in vivo*. All studies reviewed here have focused on single-species biofilms, however multi-species biofilms are also a major clinical and industrial issue (Del Pozo, 2018; Vishwakarma, 2020; Rather et al., 2021). Very few studies have been conducted investigating the dispersal of multi-species biofilms (Mei et al., 2013; Oliveira et al., 2014; Si et al., 2015; Wu et al., 2016; Ioannidis et al., 2019), and none featuring co-treatments with antibiotics. Multi-species biofilms are classically harder to eradicate than single-species biofilms given their heterogeneous composition, and research into the molecular interplay between participating species should illuminate potential therapeutic avenues (single or combination approaches) and advance the biofilm field going forward.

In most of these studies co-treatment or hybrid compounds have been much more effective in terms of biofilm inhibition and/or eradication than standalone treatments, with either the antibiofilm agent or antibiotic alone. This trend is evident across all classes of dispersal agents, suggesting that a two-pronged approach of biofilm dispersal and eradication could translate to a successful treatment strategy. Furthermore, there appears to be a distinct advantage to the simultaneous delivery and or release of the two agents at the target site, an effect which was demonstrated by the production of hybrid molecules, and delivery and release systems. However, very few cotreatments have been explored for their potential as hybrids drugs. This review has highlighted a multitude of effective cotreatments that would be ideal for future development into hybrid drugs.

Despite the advantages of some combination strategies having been demonstrated both *in vitro* and *in vivo*, follow-up *in vivo* studies are often lacking for co-treatments with confirmed anti-biofilm activity *in vitro*. Indeed, out of the 45 studies reviewed, only 17 (~38%) progressed from *in vitro* testing to preclinical evaluation in an *in vivo* model. Furthermore, out of the hundreds of newly discovered antibiofilm agents currently reported in the literature, only a handful have been tested for activity *in vitro* in assays comparing single agents over co-treatment. This highlights that there is still a lot of untapped potential for the development of new effective anti-biofilm combination therapies. Future studies on novel antibiofilm agents should follow a co-treatment design from early testing, first examining the agent’s antibiotic potentiation activity *in vitro* and if successful progress into *in vivo* testing in relevant animal models. While this process can appear tedious, it is necessary in order to shortlist promising combination leads for clinical development. We posit that this review has provided examples that are already following this process successfully and identified others that represent promising candidates to follow this path.

Of the agents reviewed here, QSIs, NO, and nitroxides appear to hold the most promise for the development of biofilm-eradication treatments based on the co-treatment strategy. While the efficacy of co-treatments using QSIs has been demonstrated *in vivo* in some species, both NO and nitroxides remain to be examined *in vivo* despite their promising *in vitro* results. Any of these candidates would be ideal to examine in *in vivo* models.

Biofilm-related infection remains a critical healthcare issue worldwide, and new and innovative strategies need to be devised. Co-treatment using antibiofilm agents with antibiotics appears to hold great promise as one such strategy, and our review provides useful information required to progress and assist in the development of candidates along this strategy.

## Author Contributions

AV and MT contributed to conception and design of the review. SH and AV compiled and reviewed relevant studies. SH wrote the first draft of the manuscript, figures, and tables. AV and MT wrote sections of the manuscript and edited tables. All authors contributed to the article and approved the submitted version.

## Funding

SH is the recipient of an Australian Government Research Training Program (RTP) Scholarship. 

## Conflict of Interest

The authors declare that the research was conducted in the absence of any commercial or financial relationships that could be construed as a potential conflict of interest.

## Publisher’s Note

All claims expressed in this article are solely those of the authors and do not necessarily represent those of their affiliated organizations, or those of the publisher, the editors and the reviewers. Any product that may be evaluated in this article, or claim that may be made by its manufacturer, is not guaranteed or endorsed by the publisher.
