# Supplementary material for: Combination Therapies for Biofilm Inhibition and Eradication: A Comparative Review of Laboratory and Preclinical Studies
Source: Front Cell Infect Microbiol. 2022 Feb 25;12:850030. doi: 10.3389/fcimb.2022.850030 (PMC8915430; doi:10.3389/fcimb.2022.850030)
Supplement: Supplementary file 1 [file Table_1.docx]

**Table S1. List of all studies involving combination treatments against bacterial biofilms currently in development**. All studies investigating biofilm co-treatments cited in this review were compiled into this table, which lists the efficacy of the dispersal agent, antibiotic alone, and combination treatment. These studies are summarized here under different dispersal agent groups (QSIs, NO/Nitroxides, AMPs, Repurposed Drugs), as they appear in the main text. Other study details provided include the name of the dispersal agent(s) and antibiotic(s), bacterial species tested, if the combination treatment was tested against biofilm infection *in vivo*, and if it was tested for inhibition, dispersal and/or eradication of the target biofilm*.* Efficacy measures for each study are different and are described in the “Efficacy parameter” column; efficacy measures reported are in relation to untreated biofilm controls in most studies, unless otherwise specified.

| **Dispersal Agent** | **Antibiotic** | **Co-treatment/**  **hybrid** | **Tested Species** | **Efficacy parameter** | **Compared to untreated biofilm (unless specified otherwise)** | | | ***In vitro*** | ***In vivo*** | **Inhibiting biofilm formation** | **Eradication/Dispersal** | **Ref** |
| --- | --- | --- | --- | --- | --- | --- | --- | --- | --- | --- | --- | --- |
|  |  |  |  |  | **Combination efficacy** | **Efficacy of dispersal agent alone** | **Efficacy of antibiotic alone** |  |  |  |  |  |
| **Quorum Sensing Inhibitors (QSIs)** | | | | | | | | | | | | |
| Baicalin hydrate, cinnamaldehyde, hamamelitannin | Tobramycin, clindamycin, vancomycin | Co-treatment | *P. aeruginosa, S. aureus* | CFU count (Mueller-Hinton Agar) | 68-90% decrease (*P. aeruginosa),* | No significant decrease | 6% (*P. aeruginosa* ATCC 9027) 45% (*P. aeruginosa* PAO1) | ✓ |  |  | ✓ | [1] |
|  |  |  |  |  | 6% decrease  *(S. aureus* Mu50) |  |  |  |  |  |  |  |
|  |  |  |  |  |  |  | 2-13% (*S. aureus* CS1/Mu50) decrease |  |  |  |  |  |
| Baicalin hydrate, cinnamaldehyde, hamamelitannin | Tobramycin, clindamycin, vancomycin | Co-treatment | *P. aeruginosa, B. cenocepacia, S. aureus* | Survival assay (% survival after 48h), CFU count (mouse) | *C. elegans*  65-75% (*B. cenocepacia*), 80-90% (*P. aeruginosa*), 80% (*S. aureus*) | *C. elegans*  None/minimal effect | *C. elegans*  38-55% (*B. cenocepacia*), 62-65% (*P. aeruginosa*), 52% (*S. aureus*) |  | ✓ (nematode, moth larvae, mouse) |  | ✓ | [1] |
|  |  |  |  |  | *G. mellonella*  40% (*B. cenocepacia/B. multivorans*), 90-100% (*S. aureus*) | *G. mellonella*  0-30% (*B. cenocepacia/B. multivorans*), 70-100% (*S. aureus*) | *G. mellonella*  10-25% (*B. cenocepacia/B. multivorans*), 80-90% (*S. aureus*) |  |  |  |  |  |
|  |  |  |  |  | Mouse (2 mg/kg baicalin hydrate & 10/20/30 mg/kg tobramycin)  52.1% reduction (10), 85.9% (20), 99% (30) |  | Mouse  47.5% reduction (10 mg/kg), 88.8% (20 mg/kg), 91.9% (30 mg/kg) |  |  |  |  |  |
| Furanone C-30, ajoene, horseradish extract | Tobramycin | Co-treatment | *P. aeruginosa* | CFU Count (1,2,3,14 days post infection) | **C-30:** 1-log reduction  **Ajoene:** 2-log reduction (3 days post), <1-log reduction (14 days post, one mouse cleared abcess entirely with a 4-log-reduction)  **Horseradish:** <1- to 3-log reduction (3 & 14 days post) | **C-30** (2 days post): <1-log reduction  **Ajoene**:1-2 log reduction (3 days post), <1-log reduction (14 days post)  **Horseradish:** <1-log reduction (3 & 14 days post) | ≤1-log reduction (all test parameters) |  | ✓ (mouse) |  | ✓ | [2] |
| FS3 | Daptomycin | Co-treatment | *S. aureus* | CFU Count (7 days post implantation) | 5-log reduction | 2-log reduction | 3-log reduction |  | ✓ (mouse) |  | ✓ | [3] |
| FS8 | Tigecycline | Co-treatment | *S. aureus* | CFU Count (7 days post implantation) | 5-log reduction | 2-log reduction | 2-log reduction |  | ✓ (mouse) |  | ✓ | [4] |
| C11 | Ciprofloxacin, tobramycin, ceftazidime, colistin | Co-treatment | *P. aeruginosa* | Antibiofilm assay (log CFU/cm^2^) | 1-2 log reduction (ceftazidime) | <1-log to 2-log reduction | 1-2-log reduction | ✓ |  |  | ✓ | [5] |
|  |  |  |  |  | 4-6-log reduction (ciprofloxacin, tobramycin, colistin) |  |  |  |  |  |  |  |
| Hamamelitannin | vancomycin, cefazolin, cefalonium, cephalexin, cefoxitin, daptomycin, linezolid, tobramycin, fusidic acid | Co-treatment | *S. aureus* | CFU Count (% eradication) | VAN: 65%  CZ: 90%  CL: 60%  CFL: 70%  Cfx: 90%  DAP: 70%  LNZ: 30%  TOB: 90%  FA: 95% | - | VAN: 35%  CZ: 65%  CL: 15%  CFL: <1%  Cfx: 65%  DAP: 15%  LNZ: <1%  TOB: 50%  FA: 95% | ✓ |  |  | ✓ | [6] |
| Cyclodextrin– Hamamelitannin | Vancomycin | Co-treatment | *S. aureus* | CFU Count | 5.5-log reduction | 1-log reduction | 3.5-log reduction | ✓ |  |  | ✓ | [7] |
| FS10 | Tigecycline | Co-treatment, Allevyn foam wound dressing | *S. aureus* (MSSA and MRSA) | CFU Count (7 days post implantation) | MSSA: 6-log reduction  MRSA: 5.75-log reduction | MSSA: 1.75-log reduction  MRSA: 1.5-log reduction | MSSA: 4-log reduction  MRSA: 3.5-log reduction |  | ✓(mouse) |  | ✓ | [8] |
| Hamamelitannin analogue 38 | Vancomycin, cefazolin, cefalonium, cephalexin, daptomycin, linezolid, tobramycin | Co-treatment | *S. aureus* | % eradication  VAN, DAP: 20 µg/mL  CZ, CL, CFL: 1000 µg/mL  LNZ: 20 µg/mL  TOB: 1024 µg/mL  hamamelitannin analogue 38: 100 µM | VAN: 90%  CZ: 95%  CL: 65%  CFL: 55%  DAP: 95%  LNZ: 65%  TOB: 95% | - | VAN: 35%  CZ: 75%  CL: 30%  CFL: 30%  DAP: 20%  LNZ: 15%  TOB: 65% | ✓ |  |  | ✓ | [9] |
| Hamamelitannin analogue 38 | Vancomycin, cephalexin | Co-treatment | *S. aureus* Mu50 and Newbould 305 | CFU Count, Mu50 (*C. elegans*), Newbould 305 (mouse) | *C. elegans:* 1.75- log reduction  Mouse: 5.75-log reduction | *C. elegans:* 1-log reduction  Mouse: <1-log reduction | *C. elegans:* 0.5-log reduction  Mouse: 3.75-log reduction |  | ✓(nematode, mouse) |  | ✓ | [9] |
| Baicalin hydrate | Tobramycin, gentamicin, kanamycin, neomycin | Co-treatment | *B. cenocepacia, B. multivorans, B. ambifaria* | Number of strains with significantly reduced CFU/mL, compared to antibiotic treatment alone | TOB: 5/9, 75-95% reduction  GEN: 4/9, 70-96%  KAN: 1/9, 98% reduction  NEO: 4/9, 50-98% reduction | - | - | ✓ |  |  | ✓ | [10] |
| 3-amino-7-chloro-2-nonylquinazolin-4(3H)-one (ACNQ) | Ciprofloxacin | Co-treatment, triethylene glycol alginate polymeric nanoparticle delivery | *P. aeruginosa* | % dead bacteria (LIVE/DEAD® BacLight™ fluorescent assay) | 80% (4 µg/mL ACNQ + 60 µg/mL ciprofloxacin) | 10% (4 µg/mL) | 50% (60 µg/m L) | ✓ |  |  | ✓ | [11] |
| Cinnamic acid derivatives: 4-dimethylaminocinnamic acid (DCA) and 4-methoxycinnamic acid (MCA) | Tobramycin | Co-treatment | *C. violaceum* | CFU Count | >5-log reduction | <1-log reduction | ≤1-log reduction | ✓ |  |  | ✓ | [12] |
| Alkylquinolone quorum sensing inhibitor (QSI (1)) | Tobramycin | Co-treatment, squalenyl hydrogen sulfate nanoparticle delivery | *P. aeruginosa* | CFU Count 25 µg/mL (tobramycin), 20 µM (QSI (1)) | Without nanoparticle: 4-5-log reduction  With nanoparticle: >8-log reduction (99.9% eradication) | - | Without nanoparticle: 4-5-log reduction  With nanoparticle: >5-log reduction | ✓ |  |  | ✓ | [13] |
| Baicalin | Levofloxacin, amikacin, ceftazidime | Co-treatment | *P. aeruginosa* | CFU Count  Baicalin: 128 ug/mL  LEV: 1 ug/mL  AMK: 8 ug/mL  CAZ: 2ug/mL | LEV: 2-log reduction  AMK: 3.5-log reduction  CAZ: 2.5-log reduction | - | LEV: 1.5-log reduction  AMK: 2-log reduction  CAZ: 1.75-log reduction | ✓ |  |  | ✓ | [14] |
| Baicalin | Levofloxacin, amikacin, ceftazidime | Co-treatment | *P. aeruginosa* | CFU Count | LEV: 1.5-log reduction  AMK: 2-log reduction  CAZ: 2.5-log reduction | 1-log reduction | 1-log reduction |  | ✓(mouse) |  | ✓ | [14] |
| **Nitric Oxide and Nitroxides** | | | | | | | | | | | | |
| NO (sodium nitroprusside) | Tetracycline | Sequential treatment | *V. cholerae* | Biofilm surface area | 90% reduction | 67% reduction | 21% reduction | ✓ |  |  | ✓ | [15] |
| NO (diethylamin-cephalosporin-30diazeniumdiolate) | Tobramycin | Co-treatment | *P. aeruginosa* | Biofilm biomass | 65% reduction | 50% reduction | <1% reduction | ✓ |  |  | ✓ | [16] |
| NO (diazeniumdiolate nanoparticles) | Gentamicin | Co-treatment | *P. aeruginosa* | Biofilm viability | 90% reduction | ≤30% reduction | ≤30% reduction | ✓ |  |  | ✓ | [17] |
| NO (chitosan-graft-poly(amidoamine) dendrimer) | Methicillin | Co-treatment | *S. aureus* | Biofilm biomass (1 mg/mL treatment) | 85% reduction | 67% reduction | 15% reduction | ✓ |  |  | ✓ | [18] |
| NO (chitosan-graft-poly(amidoamine) dendrimer) | Methicillin | Co-treatment | *S. aureus* | MRSA viability | 99% reduction | 80% reduction | 35% reduction |  | ✓(rat) |  | ✓ | [18] |
| nitroxide 4-carboxy-2,2,6,6tetramethylpiperidine 1-oxyl (CTEMPO) | Ciprofloxacin | Co-treatment | *P. aeruginosa;* enterohemorrhagic *E. coli* | Biofilm eradication | 99.3% (*P. aeruginosa*); 87% (*E. coli*) eradication | 60% (*P. aeruginosa*); 71% (*E. coli*) eradication | <1% eradication | ✓ |  |  | ✓ | [19] |
| ciprofloxacin-nitroxide hybrid-27 | Ciprofloxacin | Hybrid | *P. aeruginosa* | Biofilm eradication | 94% eradication | - | - | ✓ |  |  | ✓ | [20] |
| CTEMPO, ciprofloxacin-CTMIO hybrid | Ciprofloxacin | Co-treatment and hybrid | *S. aureus* | Biofilm eradication (MBEC) value | 256 µM (CTEMPO co-treatment at 8 µM); 64 µM (hybrid) | >1200 µM | 4096 µM | ✓ |  |  | ✓ | [21] |
| Dinitroxide-ciprofloxacin | Ciprofloxacin | Hybrid | Uropathogenic *E. coli* | Biofilm eradication (MBEC) | 2.5-fold improvement (400 µM) | >1000 µM (CTEMPO) | 1000 µM | ✓ |  |  | ✓ | [22] |
| **Antimicrobial Peptides** | | | | | | | | | | | | |
| G10KHc | Tobramycin | Co-treatment | *P. aeruginosa* | Colony forming unit (CFU) count | 4-log reduction (4 hrs), complete eradication (24 hrs) | Minimal reduction (4 hrs & 24 hrs of treatment) | Minimal reduction (4 hrs & 24 hrs of treatment) | ✓ |  |  | ✓ | [23] |
| Tachyplesin III | Piperacillin-tazobactam | Co-treatment | *P. aeruginosa* | CFU count | 5-log reduction | 3-log reduction | 3-log reduction |  | ✓ (mouse) |  | ✓ | [24] |
| BMAP-28 | Vancomycin | Co-treatment | *E. faecalis* and *S. aureus* | Log reduction (both species) | 6-log reduction | 2-3-log reduction | 2-3-log reduction | ✓ | ✓ (mouse) |  | ✓ | [25] |
| Lactoferrin | Ciprofloxacin | Co-treatment | *P. gingivalis* | Biofilm biomass | 80% reduction | 50% reduction | 40% reduction | ✓ |  |  | ✓ | [26] |
| LFchimera | Doxycycline | Co-treatment | *A. actinomycetemcomitans* | Bacterial adhesion | 87% reduction | 6% reduction | 3% reduction | ✓ |  |  | ✓ | [26] |
| Nisin | Penicillin, chloramphenicol, ciprofloxacin | Co-treatment | *E. faecalis* | Confocal microscopy (visual), antibiotics (512 mg/L), nisin (400 U/mL) | All combinations show extensive killing of biofilm cells | Minimal biofilm eradication | Minimal-some eradication | ✓ |  |  | ✓ | [27] |
| Nisin | Polymyxin and Colistin | Co-treatment | *P. aeruginosa* | Inhibition of growth (24 hours, OD 600nm) | No growth at 1/4x MIC nisin with 1/2x - 1/5x MIC polymyxin B or colistin | Minimal inhibition | Minimal inhibition | ✓ |  | ✓ |  | [28] |
| Temporin A (TEMP-A), citropin 1.1 (CIT-1.1) and tachyplesin I linear analogue (TP-1-L) | Colistin | Co-treatment | *P. aeruginosa* and *S. aureus* | MBC (mg/L); fractional bactericidal concentration index (FBCI) | *P. aeruginosa*:  5.7 -6.3 (all synergistic) | *P. aeruginosa*: >128 (TEMP-A), >128 (CIT-1.1), 32 (TP-1-L)  *S. aureus*: 32 (TEMP-A), 32 (CIT-1.1), 32 (TP-1-L) | *P. aeruginosa*: 4  *S. aureus*: >128 | ✓ |  | ✓ |  | [29] |
|  |  |  |  |  | *S. aureus:* 2.9-4.9 (all additive and/or synergistic |  |  |  |  |  |  |  |
| Temporin A (TEMP-A), citropin 1.1 (CIT-1.1) and tachyplesin I linear analogue (TP-1-L) | Colistin | Co-treatment | *P. aeruginosa* and *S. aureus* | CFU count (24 hr established biofilm) | *P. aeruginosa*:  6-log reduction (total eradication) (Colistin + TP-1-L) | *P. aeruginosa*  ≤1-log reduction  *S. aureus*  TEMP-A/CIT-1.1: 1-2-log reduction  TP-1-L: 1-4-log reduction | *P. aeruginosa*:  1-2-log reduction  *S. aureus*:  1-1.5-log reduction | ✓ |  |  | ✓ | [29] |
|  |  |  |  |  | *S. aureus*:  1-1.5-log reduction (Colistin + TP-1-L) |  |  |  |  |  |  |  |
| indolicidin, CAMA (cecropin (1-7)–melittin A (2-9) amide), nisin | Daptomycin, linezolid, teicoplanin, ciprofloxacin, azithromycin | Co-treatment | methicillin-resistant *S. aureus* (MRSA) | MBEC (mg/L); FIC index | 11/15 synergistic, 4/15 indifferent | 640 mg/L | 512-5120 mg/L | ✓ |  | ✓ | ✓ (attachment) | [30] |
| Cecropin A | Nalidixic acid | Co-treatment | Uropathogenic *E. coli* | Crystal violet assay | 70% inhibition, 50% eradication | 73% inhibition, 50% eradication | 17% inhibition, 22% eradication | ✓ | ✓ (moth larvae, survival) | ✓ | ✓ | [31] |
| CSM5-K5 | oxacillin, meropenem, streptomycin | Co-treatment | *S. aureus, E. faecalis,* uropathogenic *E. coli* | MBEC assay (pre-formed biofilm, 3-4 hour treatment exposure) | Log reduction at sub-MIC: 2-4.5 (*S. aureus*), 3 (*E. faecalis*) (>99.8% eradication), 3.8-5.2 (*E. coli*) (>99% eradication) | Log reduction at 1 x MIC: 3 (*S. aureus*), 2 (*E. faecalis*), 4 (*E. coli*) | Log reduction at sub-MIC: ~1 | ✓ |  |  | ✓ | [32] |
| CSM5-K5 | oxacillin, meropenem, streptomycin | Co-treatment | *S. aureus*, *E. faecalis*, uropathogenic *E. coli* | CFU count (24h post infection treatment, 4-5 hour post-treatment enumeration) | Log reduction at sub-MIC: 1 (*S. aureus*), 1 (*E. faecalis*), 2 (*E. coli*) | Log reduction at 1 x MIC: 1 (*S. aureus*), 1 (*E. faecalis*), 3 (*E. coli*) | - |  | ✓ (mouse) |  |  | [32] |
| Melimine, Mel4 | Ciprofloxacin | Co-treatment | *P. aeruginosa* (ciprofloxacin resistant and sensitive isolates) | % reduction of biofilm mass at 1x MIC | Resistant:  61-66%  Sensitive: 84-90% | Resistant and sensitive: <1% | Resistant: <1%  Sensitive: 65% | ✓ |  |  | ✓ | [33] |
| Melittin (embedded in agarose-based hydrogel) | Tobramycin | Co-treatment | *P. aeruginosa* | Bioluminescence | 4.2-fold reduction | No reduction | 1.8-fold reduction |  | ✓ (mouse) |  | ✓ | [34] |
| xylitol and ethylenediaminetetraacetic acid (EDTA) embedded in a hydrogel | Gentamicin | Co-treatment | *S. aureus, B. subtilis, P. aeruginosa, and E. coli* | Biofilm metabolic activity (XTT) assay | 35-50% inhibition | 30-40% inhibition | 25-35% inhibition | ✓ |  |  | ✓ | [35] |
| 1018 | Ciprofloxacin, ceftazidime, imipenem, tobramycin | Co-treatment | ESKAPE pathogens^[[1]](#footnote-1)^ | Confocal microscopy | Eradication: Minor (*S. enterica*), Moderate (*P. aeruginosa, E. coli*), Major/Complete (*K. pneumoniae, S. aureus, A. baumannii*) | Eradication: decreased thickness, minor cell death | Eradication: minimal (100x MIC ciprofloxacin against *P. aeruginosa*) | ✓ |  | ✓ | ✓ | [36] |
| DJK-5, DJK-6 | ceftazidime, ciprofloxacin, imipenem, tobramycin | Co-treatment | *P. aeruginosa,* enterohemorrhagic *E. coli* | MBIC_50_ (50% biofilm inhibition), Survival assay, confocal microscopy | *In vitro*  **Inhibition:** 2-64-fold decrease in antibiotic required for MBIC_50_  **Eradication:**  Major/Complete (DJK-5), Minimal (DJK-5, *E. coli*), Moderate (DJK-6) | *In vitro*  Inhibition: 0.8-8 µg/ml  *In vivo*  Survival: 90-96% (*C. elegans*) 30-42% (*G. mellonella*) 48 hours post infection | - | ✓ | ✓ (nematode, moth larvae) | ✓ | ✓ | [37] |
| AMP38 | Imipenem | Co-treatment | *P. aeruginosa* | MBEC | 62.5 µg/mL | >500 µg/mL | >500 µg/mL | ✓ |  |  | ✓ | [38] |
| WLBU2 | Imipenem, tobramycin, amoxicillin-clavulanate, ciprofloxacin | Co-treatment | *A. baumanii* | MBEC | 3.4-27.2 µg/mL | 85-680 µg/mL | - | ✓ |  |  | ✓ | [39] |
| 1002, HHC-10, 1018, DJK-5 | Ciprofloxacin, clindamycin colistin, erythromycin, gentamicin, meropenem, vancomycin | Co-treatment | ESKAPE pathogens1 | Abcess wound model (CFU count) | **DJK-5**  *P. aeruginosa* (245-fold with ciprofloxacin), *E. faecium* (265-fold with gentamicin), *K. pneumoniae* (91-fold with meropenem), *A. baumannii* (1325-fold with erythromycin, 2006-fold with meropenem), *S. aureus* (11-fold with vancomycin) | **DJK-5**  *P. aeruginosa* (4.6-fold), *E. faecium* (22-fold), *K. pneumoniae* (4.0-fold), *A. baumannii* (9.9-fold), and *E. coli* (2.2-fold)  **1002, 1018, HHC-10**  <1-fold, visually reduced abscess size | **Meropenem**  *A. baumannii* (111-fold), *K. pneumoniae* (2.7-fold)  **Ciprofloxacin**  *P. aeruginosa* (15-fold), *E. coli* (5.8-fold), *E. cloacae* (2.8-fold)  **Gentamicin**  *E. faecium* (4-fold)  **Clindamycin/Vancomycin**  *S. aureus* (<1-fold, visually reduced abscess size) |  | ✓(mouse) |  | ✓ | [40] |
| **D-Amino Acids** | | | | | | | | | | | | |
| D-amino acids | clindamycin, cefazolin, oxacillin, rifampin, vancomycin, amikacin, colistin, ciprofloxacin, imipenem, ceftazidime | Co-treatment | *S. aureus, P. aeruginosa* | MBIC, CFU count (24 hour established biofilm) | Inhibition: 8-1024 µg/mL  Eradication: 2-4 log reduction (*S. aureus*), 1.5-3 log reduction (*P. aeruginosa*) | - | Inhibition: 32-1024 µg/mL  Eradication: ~1-2 log reduction (*S. aureus*), ~1 log reduction (*P. aeruginosa*) | ✓ |  | ✓ | ✓ | [41] |
| D-aspartic acid, D-glutamic acid | ciprofloxacin | Co-treatment | *S. aureus* | Crystal violet assay, CFU count | Inhibition: 96.89% (40 mM, 1/4/8x MBC)  Dispersal: 37.55% (40 mM, 1/4/8x MBC) | Inhibition: 96.89% (40 mM)  Dispersal: 37.55% (40 mM) | Inhibition: 92% (8x MBC)  Dispersal: 25% (8x MBC) | ✓ |  | ✓ | ✓ | [42] |
| **Repurposed Drugs** | | | | | | | | | | | | |
| *N*-acetyl cysteine | ampicillin, ciprofloxacin, tetracycline, metronidazole | Co-treatment | *P. intermedia* | Bacterial viability assay (SYTO-9, propidium iodide) | Eradication: 40-60% (1x MIC) | Inhibition: 66% (1x MIC)  Eradication: 10-20% (1x MIC) | Eradication: 60-80% | ✓ |  | ✓ | ✓ | [43] |
| Ambroxol | Vancomycin | Co-treatment | *S. epidermidis* | Biofilm thickness, rabbit intravenous model (CFU count after 3 days treatment) | *In vitro*  60% reduction  *In vivo*  7-log reduction | *In vitro*  25% reduction  *In vivo*  <1 log reduction | *In vitro*  <1% reduction  *In vivo*  ~3-log reduction | ✓ | ✓(rabbit) | ✓ | ✓ | [44] |
| Auranofin | Fosfomycin, linezolid, chloramphenicol | Co-treatment | *S. aureus, E. faecalis* | MBEC­_50_ (mg/L) | *S. aureus:* 8-16 (LZD), 16-64 (FOF)  *E. faecalis:* 2-8 (CHL) | *S. aureus:* 16  *E. faecalis:* 8 | *S. aureus:* >128 (LZD), >64 (FOF)  *E. faecalis:* 64 (CHL) | ✓ |  |  | ✓ | [45] |
| Auranofin | Fosfomycin, linezolid, chloramphenicol | Co-treatment | *S. aureus, E. faecalis* | CFU Count per abscess (2 days post infection) | *S. aureus:* 3-4.5-log reduction (LZD), 3-5-log reduction (FOF)  *E. faecalis:* 1-log reduction (CHL) | <1-log reduction | *S. aureus: ≤*1-log reduction (LZD), 1-1.5-log reduction (FOF)  *E. faecalis:* <1-log reduction (CHL) |  | ✓ (mouse) |  | ✓ | [45] |

1. Brackman, G.; Cos, P.; Maes, L.; Nelis, H.J.; Coenye, T. Quorum sensing inhibitors increase the susceptibility of bacterial biofilms to antibiotics in vitro and in vivo. *Antimicrob Agents Chemother* **2011**, *55*, 2655-2661, doi:10.1128/aac.00045-11.

2. Christensen, L.D.; van Gennip, M.; Jakobsen, T.H.; Alhede, M.; Hougen, H.P.; Høiby, N.; Bjarnsholt, T.; Givskov, M. Synergistic antibacterial efficacy of early combination treatment with tobramycin and quorum-sensing inhibitors against Pseudomonas aeruginosa in an intraperitoneal foreign-body infection mouse model. *J Antimicrob Chemother* **2012**, *67*, 1198-1206, doi:10.1093/jac/dks002.

3. Cirioni, O.; Mocchegiani, F.; Cacciatore, I.; Vecchiet, J.; Silvestri, C.; Baldassarre, L.; Ucciferri, C.; Orsetti, E.; Castelli, P.; Provinciali, M., et al. Quorum sensing inhibitor FS3-coated vascular graft enhances daptomycin efficacy in a rat model of staphylococcal infection. *Peptides* **2013**, *40*, 77-81, doi:10.1016/j.peptides.2012.12.002.

4. Simonetti, O.; Cirioni, O.; Mocchegiani, F.; Cacciatore, I.; Silvestri, C.; Baldassarre, L.; Orlando, F.; Castelli, P.; Provinciali, M.; Vivarelli, M., et al. The efficacy of the quorum sensing inhibitor FS8 and tigecycline in preventing prosthesis biofilm in an animal model of staphylococcal infection. *Int J Mol Sci* **2013**, *14*, 16321-16332, doi:10.3390/ijms140816321.

5. Furiga, A.; Lajoie, B.; El Hage, S.; Baziard, G.; Roques, C. Impairment of Pseudomonas aeruginosa Biofilm Resistance to Antibiotics by Combining the Drugs with a New Quorum-Sensing Inhibitor. *Antimicrob Agents Chemother* **2015**, *60*, 1676-1686, doi:10.1128/aac.02533-15.

6. Brackman, G.; Breyne, K.; De Rycke, R.; Vermote, A.; Van Nieuwerburgh, F.; Meyer, E.; Van Calenbergh, S.; Coenye, T. The Quorum Sensing Inhibitor Hamamelitannin Increases Antibiotic Susceptibility of Staphylococcus aureus Biofilms by Affecting Peptidoglycan Biosynthesis and eDNA Release. *Sci Rep* **2016**, *6*, 20321, doi:10.1038/srep20321.

7. Brackman, G.; Garcia-Fernandez, M.J.; Lenoir, J.; De Meyer, L.; Remon, J.P.; De Beer, T.; Concheiro, A.; Alvarez-Lorenzo, C.; Coenye, T. Dressings Loaded with Cyclodextrin-Hamamelitannin Complexes Increase Staphylococcus aureus Susceptibility Toward Antibiotics Both in Single as well as in Mixed Biofilm Communities. *Macromol Biosci* **2016**, *16*, 859-869, doi:10.1002/mabi.201500437.

8. Simonetti, O.; Cirioni, O.; Cacciatore, I.; Baldassarre, L.; Orlando, F.; Pierpaoli, E.; Lucarini, G.; Orsetti, E.; Provinciali, M.; Fornasari, E., et al. Efficacy of the Quorum Sensing Inhibitor FS10 Alone and in Combination with Tigecycline in an Animal Model of Staphylococcal Infected Wound. *PLoS One* **2016**, *11*, e0151956, doi:10.1371/journal.pone.0151956.

9. Vermote, A.; Brackman, G.; Risseeuw, M.D.; Vanhoutte, B.; Cos, P.; Van Hecke, K.; Breyne, K.; Meyer, E.; Coenye, T.; Van Calenbergh, S. Hamamelitannin Analogues that Modulate Quorum Sensing as Potentiators of Antibiotics against Staphylococcus aureus. *Angew Chem Int Ed Engl* **2016**, *55*, 6551-6555, doi:10.1002/anie.201601973.

10. Slachmuylders, L.; Van Acker, H.; Brackman, G.; Sass, A.; Van Nieuwerburgh, F.; Coenye, T. Elucidation of the mechanism behind the potentiating activity of baicalin against Burkholderia cenocepacia biofilms. *PLoS One* **2018**, *13*, e0190533, doi:10.1371/journal.pone.0190533.

11. Singh, N.; Romero, M.; Travanut, A.; Monteiro, P.F.; Jordana-Lluch, E.; Hardie, K.R.; Williams, P.; Alexander, M.R.; Alexander, C. Dual bioresponsive antibiotic and quorum sensing inhibitor combination nanoparticles for treatment of Pseudomonas aeruginosa biofilms in vitro and ex vivo. *Biomater Sci* **2019**, *7*, 4099-4111, doi:10.1039/c9bm00773c.

12. Cheng, W.J.; Zhou, J.W.; Zhang, P.P.; Luo, H.Z.; Tang, S.; Li, J.J.; Deng, S.M.; Jia, A.Q. Quorum sensing inhibition and tobramycin acceleration in Chromobacterium violaceum by two natural cinnamic acid derivatives. *Appl Microbiol Biotechnol* **2020**, *104*, 5025-5037, doi:10.1007/s00253-020-10593-0.

13. Ho, D.K.; Murgia, X.; De Rossi, C.; Christmann, R.; Hüfner de Mello Martins, A.G.; Koch, M.; Andreas, A.; Herrmann, J.; Müller, R.; Empting, M., et al. Squalenyl Hydrogen Sulfate Nanoparticles for Simultaneous Delivery of Tobramycin and an Alkylquinolone Quorum Sensing Inhibitor Enable the Eradication of P. aeruginosa Biofilm Infections. *Angew Chem Int Ed Engl* **2020**, *59*, 10292-10296, doi:10.1002/anie.202001407.

14. Luo, J.; Dong, B.; Wang, K.; Cai, S.; Liu, T.; Cheng, X.; Lei, D.; Chen, Y.; Li, Y.; Kong, J., et al. Baicalin inhibits biofilm formation, attenuates the quorum sensing-controlled virulence and enhances Pseudomonas aeruginosa clearance in a mouse peritoneal implant infection model. *PLoS One* **2017**, *12*, e0176883, doi:10.1371/journal.pone.0176883.

15. Barraud, N.; Storey, M.V.; Moore, Z.P.; Webb, J.S.; Rice, S.A.; Kjelleberg, S. Nitric oxide-mediated dispersal in single- and multi-species biofilms of clinically and industrially relevant microorganisms. *Microb Biotechnol* **2009**, *2*, 370-378, doi:10.1111/j.1751-7915.2009.00098.x.

16. Soren, O.; Rineh, A.; Silva, D.G.; Cai, Y.; Howlin, R.P.; Allan, R.N.; Feelisch, M.; Davies, J.C.; Connett, G.J.; Faust, S.N., et al. Cephalosporin nitric oxide-donor prodrug DEA-C3D disperses biofilms formed by clinical cystic fibrosis isolates of Pseudomonas aeruginosa. *J Antimicrob Chemother* **2020**, *75*, 117-125, doi:10.1093/jac/dkz378.

17. Nguyen, T.K.; Selvanayagam, R.; Ho, K.K.K.; Chen, R.; Kutty, S.K.; Rice, S.A.; Kumar, N.; Barraud, N.; Duong, H.T.T.; Boyer, C. Co-delivery of nitric oxide and antibiotic using polymeric nanoparticles. *Chem Sci* **2016**, *7*, 1016-1027, doi:10.1039/c5sc02769a.

18. Liu, S.; Cai, X.; Xue, W.; Ma, D.; Zhang, W. Chitosan derivatives co-delivering nitric oxide and methicillin for the effective therapy to the methicillin-resistant S. aureus infection. *Carbohydr Polym* **2020**, *234*, 115928, doi:10.1016/j.carbpol.2020.115928.

19. Reffuveille, F.; Fuente-Núñez Cde, L.; Fairfull-Smith, K.E.; Hancock, R.E. Potentiation of ciprofloxacin action against Gram-negative bacterial biofilms by a nitroxide. *Pathog Dis* **2015**, *73*, doi:10.1093/femspd/ftv016.

20. Verderosa, A.D.; de la Fuente-Núñez, C.; Mansour, S.C.; Cao, J.; Lu, T.K.; Hancock, R.E.W.; Fairfull-Smith, K.E. Ciprofloxacin-nitroxide hybrids with potential for biofilm control. *Eur J Med Chem* **2017**, *138*, 590-601, doi:10.1016/j.ejmech.2017.06.058.

21. Verderosa, A.D.; Dhouib, R.; Fairfull-Smith, K.E.; Totsika, M. Nitroxide Functionalized Antibiotics Are Promising Eradication Agents against Staphylococcus aureus Biofilms. *Antimicrob Agents Chemother* **2019**, *64*, doi:10.1128/aac.01685-19.

22. Verderosa, A.D.; Harris, J.; Dhouib, R.; Totsika, M.; Fairfull-Smith, K.E. Eradicating uropathogenic Escherichia coli biofilms with a ciprofloxacin-dinitroxide conjugate. *Medchemcomm* **2019**, *10*, 699-711, doi:10.1039/c9md00062c.

23. Eckert, R.; Brady, K.M.; Greenberg, E.P.; Qi, F.; Yarbrough, D.K.; He, J.; McHardy, I.; Anderson, M.H.; Shi, W. Enhancement of antimicrobial activity against pseudomonas aeruginosa by coadministration of G10KHc and tobramycin. *Antimicrob Agents Chemother* **2006**, *50*, 3833-3838, doi:10.1128/aac.00509-06.

24. Minardi, D.; Ghiselli, R.; Cirioni, O.; Giacometti, A.; Kamysz, W.; Orlando, F.; Silvestri, C.; Parri, G.; Kamysz, E.; Scalise, G., et al. The antimicrobial peptide tachyplesin III coated alone and in combination with intraperitoneal piperacillin-tazobactam prevents ureteral stent Pseudomonas infection in a rat subcutaneous pouch model. *Peptides* **2007**, *28*, 2293-2298, doi:10.1016/j.peptides.2007.10.001.

25. Orlando, F.; Ghiselli, R.; Cirioni, O.; Minardi, D.; Tomasinsig, L.; Mocchegiani, F.; Silvestri, C.; Skerlavaj, B.; Riva, A.; Muzzonigro, G., et al. BMAP-28 improves the efficacy of vancomycin in rat models of gram-positive cocci ureteral stent infection. *Peptides* **2008**, *29*, 1118-1123, doi:10.1016/j.peptides.2008.03.005.

26. Lachica, M.; Anutrakunchai, C.; Prajaneh, S.; Nazmi, K.; Bolscher, J.G.M.; Taweechaisupapong, S. Synergistic effects of LFchimera and antibiotic against planktonic and biofilm form of Aggregatibacter actinomycetemcomitans. *PLoS One* **2019**, *14*, e0217205, doi:10.1371/journal.pone.0217205.

27. Tong, Z.; Zhang, Y.; Ling, J.; Ma, J.; Huang, L.; Zhang, L. An in vitro study on the effects of nisin on the antibacterial activities of 18 antibiotics against Enterococcus faecalis. *PLoS One* **2014**, *9*, e89209, doi:10.1371/journal.pone.0089209.

28. Field, D.; Seisling, N.; Cotter, P.D.; Ross, R.P.; Hill, C. Synergistic Nisin-Polymyxin Combinations for the Control of Pseudomonas Biofilm Formation. *Front Microbiol* **2016**, *7*, 1713, doi:10.3389/fmicb.2016.01713.

29. Jorge, P.; Grzywacz, D.; Kamysz, W.; Lourenço, A.; Pereira, M.O. Searching for new strategies against biofilm infections: Colistin-AMP combinations against Pseudomonas aeruginosa and Staphylococcus aureus single- and double-species biofilms. *PLoS One* **2017**, *12*, e0174654, doi:10.1371/journal.pone.0174654.

30. Mataraci, E.; Dosler, S. In vitro activities of antibiotics and antimicrobial cationic peptides alone and in combination against methicillin-resistant Staphylococcus aureus biofilms. *Antimicrob Agents Chemother* **2012**, *56*, 6366-6371, doi:10.1128/aac.01180-12.

31. Kalsy, M.; Tonk, M.; Hardt, M.; Dobrindt, U.; Zdybicka-Barabas, A.; Cytrynska, M.; Vilcinskas, A.; Mukherjee, K. The insect antimicrobial peptide cecropin A disrupts uropathogenic Escherichia coli biofilms. *NPJ Biofilms Microbiomes* **2020**, *6*, 6, doi:10.1038/s41522-020-0116-3.

32. Thappeta, K.R.V.; Vikhe, Y.S.; Yong, A.M.H.; Chan-Park, M.B.; Kline, K.A. Combined Efficacy of an Antimicrobial Cationic Peptide Polymer with Conventional Antibiotics to Combat Multidrug-Resistant Pathogens. *ACS Infect Dis* **2020**, *6*, 1228-1237, doi:10.1021/acsinfecdis.0c00016.

33. Yasir, M.; Dutta, D.; Willcox, M.D.P. Activity of Antimicrobial Peptides and Ciprofloxacin against Pseudomonas aeruginosa Biofilms. *Molecules* **2020**, *25*, doi:10.3390/molecules25173843.

34. Maiden, M.M.; Zachos, M.P.; Waters, C.M. Hydrogels Embedded With Melittin and Tobramycin Are Effective Against Pseudomonas aeruginosa Biofilms in an Animal Wound Model. *Front Microbiol* **2019**, *10*, 1348, doi:10.3389/fmicb.2019.01348.

35. Anjum, A.; Sim, C.H.; Ng, S.F. Hydrogels Containing Antibiofilm and Antimicrobial Agents Beneficial for Biofilm-Associated Wound Infection: Formulation Characterizations and In vitro Study. *AAPS PharmSciTech* **2018**, *19*, 1219-1230, doi:10.1208/s12249-017-0937-4.

36. Reffuveille, F.; de la Fuente-Núñez, C.; Mansour, S.; Hancock, R.E. A broad-spectrum antibiofilm peptide enhances antibiotic action against bacterial biofilms. *Antimicrob Agents Chemother* **2014**, *58*, 5363-5371, doi:10.1128/aac.03163-14.

37. de la Fuente-Núñez, C.; Reffuveille, F.; Mansour, S.C.; Reckseidler-Zenteno, S.L.; Hernández, D.; Brackman, G.; Coenye, T.; Hancock, R.E. D-enantiomeric peptides that eradicate wild-type and multidrug-resistant biofilms and protect against lethal Pseudomonas aeruginosa infections. *Chem Biol* **2015**, *22*, 196-205, doi:10.1016/j.chembiol.2015.01.002.

38. Rudilla, H.; Fusté, E.; Cajal, Y.; Rabanal, F.; Vinuesa, T.; Viñas, M. Synergistic Antipseudomonal Effects of Synthetic Peptide AMP38 and Carbapenems. *Molecules* **2016**, *21*, doi:10.3390/molecules21091223.

39. Swedan, S.; Shubair, Z.; Almaaytah, A. Synergism of cationic antimicrobial peptide WLBU2 with antibacterial agents against biofilms of multi-drug resistant Acinetobacter baumannii and Klebsiella pneumoniae. *Infect Drug Resist* **2019**, *12*, 2019-2030, doi:10.2147/idr.S215084.

40. Pletzer, D.; Mansour, S.C.; Hancock, R.E.W. Synergy between conventional antibiotics and anti-biofilm peptides in a murine, sub-cutaneous abscess model caused by recalcitrant ESKAPE pathogens. *PLoS Pathog* **2018**, *14*, e1007084, doi:10.1371/journal.ppat.1007084.

41. Sanchez, C.J., Jr.; Akers, K.S.; Romano, D.R.; Woodbury, R.L.; Hardy, S.K.; Murray, C.K.; Wenke, J.C. D-amino acids enhance the activity of antimicrobials against biofilms of clinical wound isolates of Staphylococcus aureus and Pseudomonas aeruginosa. *Antimicrob Agents Chemother* **2014**, *58*, 4353-4361, doi:10.1128/aac.02468-14.

42. Warraich, A.A.; Mohammed, A.R.; Perrie, Y.; Hussain, M.; Gibson, H.; Rahman, A. Evaluation of anti-biofilm activity of acidic amino acids and synergy with ciprofloxacin on Staphylococcus aureus biofilms. *Sci Rep* **2020**, *10*, 9021, doi:10.1038/s41598-020-66082-x.

43. Moon, J.H.; Jang, E.Y.; Shim, K.S.; Lee, J.Y. In vitro effects of N-acetyl cysteine alone and in combination with antibiotics on Prevotella intermedia. *J Microbiol* **2015**, *53*, 321-329, doi:10.1007/s12275-015-4500-2.

44. Zhang, Y.; Fu, Y.; Yu, J.; Ai, Q.; Li, J.; Peng, N.; Song, S.; He, Y.; Wang, Z. Synergy of ambroxol with vancomycin in elimination of catheter-related Staphylococcus epidermidis biofilm in vitro and in vivo. *J Infect Chemother* **2015**, *21*, 808-815, doi:10.1016/j.jiac.2015.08.017.

45. She, P.; Zhou, L.; Li, S.; Liu, Y.; Xu, L.; Chen, L.; Luo, Z.; Wu, Y. Synergistic Microbicidal Effect of Auranofin and Antibiotics Against Planktonic and Biofilm-Encased S. aureus and E. faecalis. *Front Microbiol* **2019**, *10*, 2453, doi:10.3389/fmicb.2019.02453.

1. *E. faecium, S. aureus, K. pneumoniae, A. baumannii, P. aeruginosa, E. cloacae* [↑](#footnote-ref-1)
